# Effect of the Number of Phenylcarbazole Units Adorned to the Silicon Atom for High Triplet Energy with High Charge Mobility

**DOI:** 10.3390/molecules30030454

**Published:** 2025-01-21

**Authors:** Mina Ahn, Sunhee Lee, Min-Ji Kim, Jeongyoon Kim, Jina Lee, Heejun Nam, Kyung-Ryang Wee, Won-Sik Han

**Affiliations:** 1 Department of Advanced Materials Chemistry, Korea University, Sejong 30019, Republic of Korea; ama1224@korea.ac.kr (M.A.); mjkim27@outlook.com (M.-J.K.); kimupr0516@korea.ac.kr (J.K.); 2 Department of Chemistry, Seoul Women’s University, Seoul 01797, Republic of Korea; lshee5@naver.com (S.L.); jina5062@gmail.com (J.L.); hjune2000@naver.com (H.N.)

**Keywords:** charge transporting material, high hole mobility, high triplet energy

## Abstract

Increasing the number of phenylcarbazole (PC) units attached to the silicon atom in organic solid-state thin films led to a remarkable enhancement in charge mobility. Specifically, the charge mobility values exhibited an increase from 1.32 × 10^−4^ cm^2^/Vs for **3PCBP** to 4.39 × 10^–4^ cm^2^/Vs for **2MCBP**, ultimately reaching 1.16 × 10^–3^ cm^2^/Vs for **MCBP**. Notably, these enhancements were achieved while maintaining a high triplet energy of 3.01 eV. DFT calculations on the spin density distribution provided insights into the nature of the improved mobility while preserving the triplet energy. The accuracy of the DFT calculations was validated by comparing the results with experimental data from photoemission spectroscopy (PES). Mobility measurements, as contemplated by DFT, allowed for a comprehensive understanding of the factors influencing enhanced mobility while keeping the triplet energy constant. This study suggested that intramolecular charge transfers played a crucial role in reducing reorganization energy, showing an inverse dependence on the number of PCs. Consequently, it was inferred that the manipulation of PC units could effectively optimize charge transfer mechanisms, offering a promising avenue for tailoring organic thin films with improved electronic properties.

## 1. Introduction

The charge-transporting characteristics of organic molecules play a pivotal role in the functionality of molecular electronic devices [1,2] such as organic light-emitting devices (OLEDs) [3], organic photovoltaic devices (OPVs) [4,5], and perovskite solar cells [6,7]. With respect to molecular electronic devices, the most important parameter is carrier mobility, which refers to the velocity of charge drift in a unit electric field, and it is decisive in evaluating their device performances and potential applications [8]. During working processes that involve charge transport, charge recombination, or exciton dissociation procedures, higher mobility leads to a faster charge transfer process in the conducting channel and more efficient operating frequency, as well as lower energy consumption [9]. Consequently, a profound comprehension and analysis of the intricate relationship between charge mobility and the geometric structure of a molecule are imperative for the successful development of materials tailored for organic electronic devices. Numerous endeavors have been dedicated to unraveling the intricacies of charge transport phenomena, which are now elucidated as non-adiabatic charge transfer processes. In these processes, the intermolecular hopping rate is calculated employing the Marcus theory [10], where parameters like reorganization energy and transfer integrals dictate the rate [11,12,13,14,15,16,17].

Achieving high mobility necessitates a meticulous molecular design featuring small reorganization energy and high transfer integrals. It has been observed that an elongated π-conjugation length can reduce reorganization energy and augment the transfer integral by promoting charge spread along the conjugated framework and enhancing the probability of intermolecular orbital overlap [18,19,20,21,22]. However, previous findings have unveiled a distinct contribution from inorganic silicon, which effectively breaks conjugation and boosts charge mobility [23,24,25,26]. The silicon atom serves as an efficient spacer, disrupting conjugation in planar organic molecules. This innovative strategy has proven instrumental in producing high triplet energy host materials [23,27,28], opening novel pathways for the development of highly efficient blue phosphorescent organic light-emitting diodes (PHOLEDs) [29,30,31,32,33,34]. Additionally, triplet materials with spatial stereo structures have shown promise in enhancing OPV performance by leveraging long-lived triplet exciton lifetimes [35]. While the existing literature has predominantly focused on the functionality of conjugation breaking, the mechanism underlying charge transport properties has been inadequately addressed. This highlights a crucial gap in understanding that merits further exploration for a comprehensive comprehension of the synergistic effects between conjugation breaking and charge mobility enhancement in organic–inorganic hybrid materials.

This work will focus on the effect of the number of PC units adorned to the silicon atom. Carbazole-based materials are among the most studied phosphorescent blue host materials due to their wide-band energy, high triplet energy level, and good charge mobility [36,37]. However, the fabrication of organic thin films is often challenged by the small molecular size of the materials, which can result in crystallization and thermal instability, thereby compromising film stability [38]. Increasing the number of carbazole units can form morphologically stable and uniform amorphous films by extending the molecular size [39]. The specificity of chemical modification affords bulky and steric molecular configurations without a decrease in the triplet energy of the molecule [23,40]. The inclusion of silicon atoms serves two purposes: breaking conjugation to achieve a wider band gap and providing tetrahedral geometry to influence the arrangement of molecular packing [41]. The interplay between the combination of these molecular structures and charge mobility offers a promising approach to tailoring organic thin films with improved electronic properties.

In this study, we propose an innovative molecular design concept that defies conventional wisdom, aiming to achieve a harmonious balance between high charge mobility and elevated triplet energy. This unconventional approach involves harnessing the benefits of three key mechanisms: firstly, the reduction of reorganization energy resulting from intramolecular charge transfer; secondly, the enhancement of intermolecular transfer integral; and thirdly, the effective conjugation disruption induced by the incorporation of a silicon atom into the molecular structure. The intricate origins and interactions governing these fascinating properties were meticulously unraveled through a comprehensive analytical framework. This encompassed in-depth mobility measurements, and an extensive quantum chemical analysis conducted on a series of PCs characterized by the strategic integration of silicon. Through this multifaceted approach, we sought to elucidate the synergistic effects and molecular intricacies that underpin the remarkable dual enhancement of charge mobility and triplet energy in these silicon-based molecular architectures.

## 2. Results and Discussion

**Figure 1:**
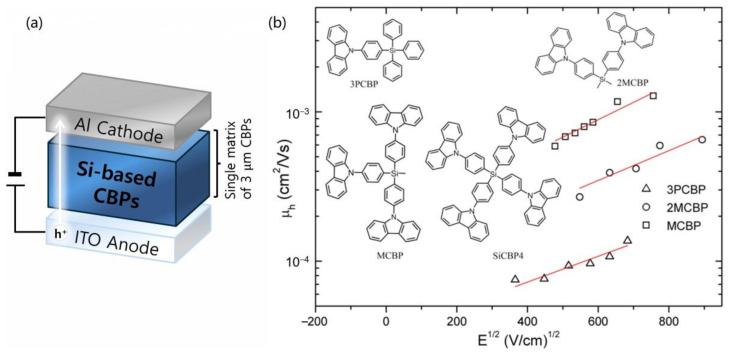
(**a**) Device architecture of hole-only device used for charge transport studies. (**b**) Chemical structures of the investigated materials and experimental drift mobility data (black shapes) and fitted curves (red lines) as a function of electric field for a series of materials with one, two, three, four PC units.

Figure 1a illustrates the device architecture of a hole-only device used for charge transport studies and the molecular structures of a series of Si-based compounds with one (**3PCBP**), two (**2MCBP**), three (**MCBP**), and four (**SiCBP4**) PC units. The photophysical properties of all compounds, including their high triplet energy, are reported to closely resemble those of the unsubstituted carbazole monomer, which can be definitively attributed to the lowest π-π* transition of the carbazole unit [23]. This result suggests that electronic interaction between the PC substituents is minimal or negligible, as the tetrahedral *sp*^3^-hybridized silicon effectively functions as a spacer, inhibiting the extension of π-conjugation from the phenyl carbazole core to the peripheral carbazole units. Moreover, the introduction of a tetrahedral silicon linkage into the PC groups not only prevented excimer formation and luminescence quenching, but also enabled the formation of thermally stable amorphous thin films [23,29].

The drift mobility data were experimentally validated as a function of the electric field using the time-of-flight (TOF) method. For this analysis, a 3 µm-thick organic (Si-based PC) thin film and an aluminum (Al) electrode were sequentially deposited onto a glass substrate pre-coated with indium tin oxide (ITO). It should be noted that the **SiCBP4** sample was excluded from this process due to its high molecular weight, which rendered thermal evaporation impractical. Specifically, the mobility values increased from 1.32 × 10^−4^, 4.39 × 10^−4^, and 1.16 × 10^−3^ cm^2^/Vs at 5 × 10^5^ V/cm for **3PCBP**, **2MCBP**, and **MCBP**, respectively. Notably, these enhancements were achieved while maintaining a high triplet energy of 3.01 eV. The experimental charge mobility data exhibits an obvious and significant increase as the number of PCs increases (Figure 1b).

**Figure 2:**
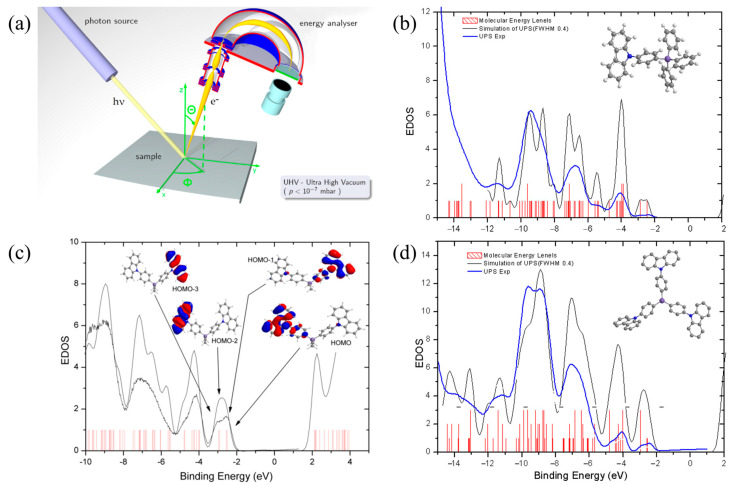
(**a**) Illustration of photoemission spectroscopy (PES) experiment setup and experimental and theoretical data for (**b**) **3PCBP**, (**c**) **2MCBP**, and (**d**) **MCBP** (inset: Atom colors are purple for Si, blue for N, and gray for C).

**Figure 3:**
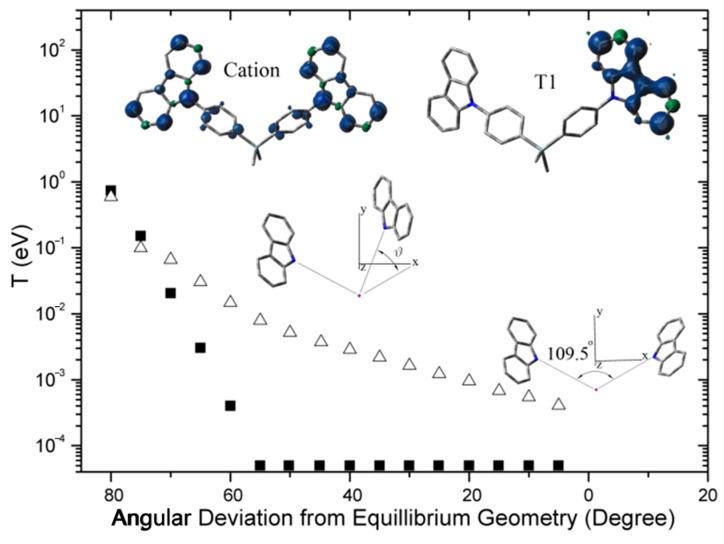
Transfer integrals for charge transfer (hollow triangles) and triplet energy transfer (filled squares) as a function of deviation angle from equilibrium position. The right PC unit is rotated counterclockwise around *z* axis (inset: spin density distribution of **2MCBP** in its cation doublet state and neutral triplet state, where the geometries were optimized for cation doublet and neutral triplet state, respectively. Positive spin density is in blue and negative spin density is in green).

To analyze the systematic increase in charge mobility with the number of PC units surrounding the Si atom, it was necessary to accurately determine the energy states of Si-based PC compounds in the film state. For this purpose, photoemission spectroscopy (PES) experiments were conducted on the films, and the results were compared with density functional theory (DFT) calculations. Based on this analysis, the optical properties and charge transport characteristics were investigated, aiming to establish a structure–property relationship grounded in molecular design. The accuracy of the calculation is confirmed by comparing the PES data to the simulated spectra obtained from the same theory and basis set [10]. From the calculation, we found that (1) the triplet energies of all the materials are almost the same not only in the calculation but also in the experiments [23], and (2) reorganization energy is inversely proportional to the number of PC units (vide infra). This similar trend is also reported by Goddard [18], where they explained the reason for reorganization energy change by the reduction of bond deformation due to increased charge delocalization with the increased number of conjugated phenyl rings. In our system, even though the conjugation is broken by the Si atom [34], the same effect is observed. Consequently, it is reasonable to deduce that when the molecule becomes charged, the charge is expected to distribute uniformly across the PCs through intramolecular hopping. The computed spin density distributions of the molecules in their cation doublet states corroborate this inference, as illustrated in Figure 3. Furthermore, analysis of the molecular orbital (MO) energies in the neutral state reveals that the highest occupied molecular orbital (HOMO) is bifurcated into two levels for **2MCBP** with a separation of 43 meV, and into three levels for **MCBP** with a separation of 27 meV, as depicted in Figure 2. The half value of these separations corresponds to the electron transfer integral, which also supports the intramolecular electron transfer (ET) [1]. On the contrary, the triplet energy is not transferred, which is evident from the experiment (Table S14) and spin density distribution of Figure 3.

To elucidate the differing behaviors, we calculated the transfer integrals for ET [1] and triplet energy transfer (TET) [42,43] in carbazole dimers across various geometrical configurations, as illustrated in the inset of Figure 3. The transfer integral for ET gradually decreases as the angle between the two carbazole units is increased, while the transfer integral for TET decreases rapidly and becomes negligible beyond an angle of 55°. These findings elucidate the complete localization of triplet energy within a single unit and the uniform distribution of excess charge across the units. As the number of carbazole units in a molecule increases, the net charge per unit correspondingly diminishes, leading to an expectation that the reorganization energy will be inversely proportional to the number of functional units within the molecule.

**Figure 4:**
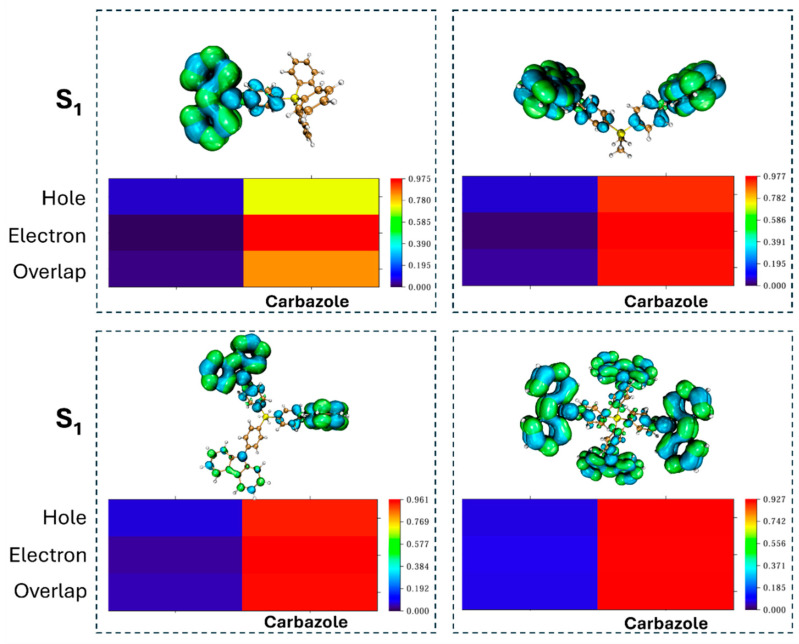
Hole–electron distributions with blue for hole and green for electron and the heat maps based on S_0_-geometry for investigated molecules.

Figure 4 shows that the holes of Si-based PC compounds are predominantly localized within the phenyl carbazole unit, while the electrons are primarily concentrated in the carbazole unit. This results in a significant overlap of holes and electrons within the carbazole unit. To quantitatively analyze the charge distribution, we partitioned all the investigated molecules into two fragments and generated contribution heat maps along with the corresponding contribution percentages for each fragment (Figure S13 and Table S13). A visual examination of the heat map colors reveals that the holes and electrons are all mainly concentrated on the carbazole unit (fragment 2) rather than the other unit (fragment 1). For example, the contributions of holes at the carbazole unit for **3PCBP**, **2MCBP**, **MCBP**, and **SiCBP4** are 72.37%, 93.41%, 92.91%, and 92.75%, respectively. Similarly, the contributions of electrons at the carbazole unit for these compounds are 92.49%, 97.71%, 96.10%, and 92.12%, respectively. Notably, as the number of carbazole units increased from one to four, the contribution percentage of holes increased while that of electrons decreased, ultimately resulting in a greater contribution from holes compared to electrons at four units. Clearly, the holes and electrons of all Si-based compounds exhibit a high degree of overlap within the carbazole unit, with overlaps exceeding 80%.

**Figure 5:**
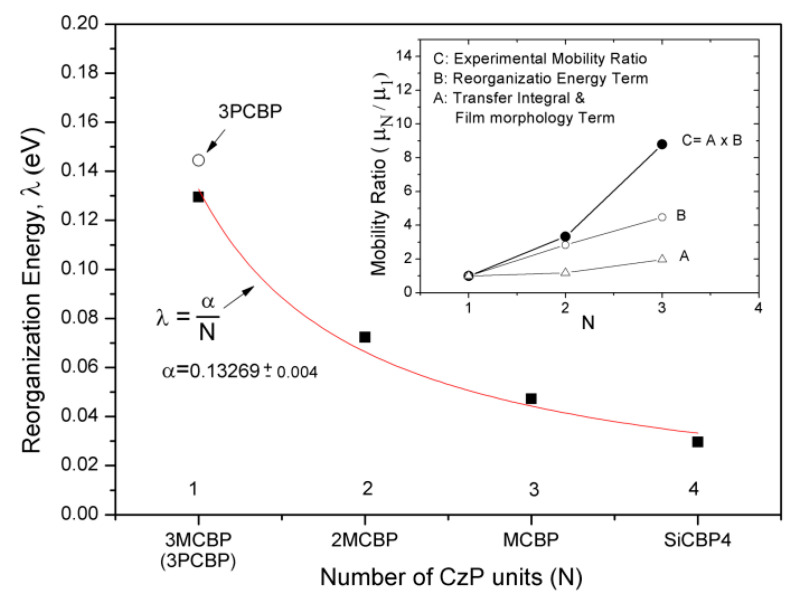
Reorganization energy as a function of the number of PC units (inset: measured mobility ratios at the electric field of 5 × 10^5^ V/cm and comparison with the ratios calculated).

Figure 5 shows the reorganization energy curves as a function of the number of units in a molecule. As expected, the calculated values of reorganization energies of **3MCBP**, **2MCBP**, **MCBP**, and **SiCBP4** can be fitted by a function *y* = 0.133/*N*. Finally, to estimate the effect of reorganization energy on the measured hole mobility and hence to derive qualitative correlation of the mobility with the intermolecular transfer integral, experimental mobility ratios are compared to calculated mobility ratios. Because of its inherent character, reorganization energy term of mobility ratio can be factored out as follows:

(1) $$\frac{\mu_{N}}{\mu_{1}}=f(t_{N}{,t}_{1})\sqrt{\frac{\lambda_{1}}{\lambda_{N}}}\exp(\frac{\lambda_{N}-\lambda_{1}}{4k_{B}T})$$

where *f* represents the complicated and unpredictable intermolecular charge transfer integral (*t*) term of amorphous film, and the other terms represent the reorganization energy term of mobility ratio [44]. The inset of Figure 5 reflects the aforementioned estimates. The primary contribution to hole mobility arises from the reorganization energy term, while the contribution from the intermolecular charge transfer integral term is comparatively minor due to the disorder present in the amorphous film. This observation aligns with findings reported in other studies [11,45]. However, the contribution from the intermolecular charge transfer integral increase significantly for **MCBP**, due to the pronounced increase in the intermolecular MO overlap, which directly results from the charge being distributed throughout the charged molecule.

Through a combination of quantum chemical calculations, TOF, and PES experiments, we confirmed the preservation of high triplet energy at the peripheries of the silicon atom, regardless of the number of structural units. Additionally, we observed a gradual increase in charge mobilities as the number of units increased. Transfer integrals for both ET and TET were systematically estimated. Our findings reveal that intramolecular charge transfer is energetically favorable, whereas intramolecular TET is prohibited. By leveraging the concepts of conjugation blocking and intramolecular charge transfer, we demonstrated the design of molecular systems capable of achieving both high triplet energy and excellent charge mobility. Notably, the silicon atom plays a dual role: it disrupts conjugation while effectively facilitating intramolecular charge transfer between the decorated PC units.

## 3. Method

### 3.1. Time-of-Flight Drift Mobility Measurement Method for Study of Charge Transport Properties of a Material

The time-of-flight (TOF) samples were prepared by sequentially depositing an organic thin film and an aluminum (Al) electrode onto a glass substrate pre-coated with indium tin oxide (ITO). The deposition process was carried out using a high-vacuum thermal evaporator. The thicknesses of both the organic and metallic layers were monitored in situ with a quartz crystal sensor, which was calibrated using scanning electron microscopy (SEM). The organic layers were deposited to a thickness of 3.0 *μ*m. The hole mobility (*μ*) as a function of the electric field was analyzed based on the Poole–Frenkel relationship, expressed as *μ* ∝ exp(b*E*^1/2^), where b is the Poole–Frenkel factor. The carrier transport characteristics remained consistent across all samples. The flight time (τ_T_) was extracted from the intersection point of two linear segments in the double-logarithmic plot of the transient photocurrent. Hole mobilities were determined at a field strength corresponding to a square root value of *E* = 500 (V/cm)^1/2^.

### 3.2. Photoemission Spectroscopy (PES) as a Probing Tool for Occupied States

For photoemission spectroscopy (PES), the organic materials were deposited onto clean polycrystalline gold foil under an ambient pressure of 5 × 10^−8^ Torr using Knudsen cell. The thickness of the deposited film was monitored with a quartz crystal microbalance (XTC2, Inficon, Bad Ragaz, Switzerland), and the thickness was pre-calibrated by the measurement of the cross-section of the film using scanning electron microscopy (SEM, HITACHI, Tokyo, Japan). The specimens were transferred to a photoemission chamber without exposure to air. UPS measurements were performed in a UHV system by using a hemispherical electron energy analyzer (SES-100, VG-SCIENTA, Saint Leonards-on-Sea, UK) with a He I (21.2 eV) gas discharge lamp with a base pressure of 5 × 10^−10^ Torr. All energy levels were referenced to the Fermi edge of the Au substrate. The spectral resolution of UPS was 100 meV, as determined from the Fermi edge of Au; the Au surface was pre-cleaned by argon ion bombardment without post-annealing. The incidence angle of the photon was 30° to the surface normal of the substrate and the photoelectron was detected at a normal angle to the substrate.

### 3.3. Density Functional Theory Calculations

Calculations were carried out using the Gaussian 16 (Revision D.03) package [46], and GaussView 5.0.9 and Chem3D 16.0 were used to visualize the results. The molecular structures determined by X-ray crystallography were used as the input geometries for optimizing their ground-state geometries without symmetry constraints. The singlet ground-state (S_0_) of **3PCBP**, **2MCBP**, **MCBP**, and **SiCBP4** was optimized at B3LYP/6-31G(d,p) [47,48,49,50,51,52]. The radical cation doublet ground-state (D_0_) of [**3PCBP**]^•+^, [**2MCBP**]^•+^, [**MCBP**]^•+^, and [**SiCBP4**]^•+^ was optimized at B3LYP/6-31G(d,p). The triplet ground-state (T_0_) of **3PCBP**, **2MCBP**, **MCBP**, and **SiCBP4** was optimized at B3LYP/6-31G(d,p). The excitation energies and oscillator strengths for the lowest 100 singlet–singlet transitions at the optimized geometry in the ground state were obtained in time-dependent DFT (TD-DFT) calculations using the same basis set and functional as for the ground state. The electron–hole analysis was analyzed using the Multiwfn 3.8 program [53].

## 4. Conclusions

The addition of PC units to the silicon atom in organic solid-state thin films resulted in a significant enhancement of charge mobility. DFT calculations on spin density distribution elucidated the mechanisms underlying the improved mobility while maintaining triplet energy levels. The reliability of the DFT calculations was confirmed through comparisons with experimental data obtained from PES. Charge mobility measurements, as informed by DFT, provided a thorough understanding of the factors contributing to increased mobility without affecting triplet energy. This study indicated that intramolecular charge transfers were essential in reducing reorganization energy, with an inverse relationship observed relative to the number of PC units. Additionally, the heightened charge distribution facilitated a greater probability of intermolecular charge transfer, further contributing to the overall enhancement in mobility. Consequently, it was concluded that strategically manipulating PC units could effectively optimize charge transfer mechanisms, presenting a promising strategy for tailoring organic thin films with enhanced electronic properties.

## Data Availability

All data are contained within the article.

## Supplementary Materials

The following supporting information can be downloaded at: https://www.mdpi.com/article/10.3390/molecules30030454/s1, General experimental information, Cartesian coordinates for optimized structures (Tables S1–S12), Frontier orbitals and orbital energy levels (Figures S1–S12), Hole and electron distribution for the S_0_→S_1_ transitions (Figure S13), Contributions of two fragments to hole, electron, and overlap (Table S13), and Physical properties (Table S14). Reference [54] is cited in the Supplementary Materials.
