# Supplementary material for: Effect of the Number of Phenylcarbazole Units Adorned to the Silicon Atom for High Triplet Energy with High Charge Mobility
Source: Molecules. 2025 Jan 21;30(3):454. doi: 10.3390/molecules30030454 (PMC11820696; doi:10.3390/molecules30030454)
Supplement: Supplementary file 1 [file molecules-30-00454-s001.zip › molecules-3429960-supplementary.pdf]

Supporting Information

Effect of the Number of Phenylcarbazole Units Adorned to the Silicon Atom for High Triplet Energy with High Charge Mobility

Mina Ahn,[1,†] Sunhee Lee,[2,†] Min-Ji Kim,[1] Jeongyoon Kim,[1] Jina Lee,[2] Heejun Nam,[2] Kyung-Ryang Wee[1,*] and Won-Sik Han[2,*]

[1]*Department of Advanced Materials Chemistry, Korea University, Sejong 30019, South Korea.*

[2]*Department of Chemistry, Seoul Women's University, 621 Hwarang-ro, Nowon-gu, Seoul 01797, South Korea.*

[†]These authors contributed equally to this work.

Table S1. Cartesian coordinates for optimized structure for **3PCBP-S$_0$**.

Symbolic Z-matrix:
Charge = 0 Multiplicity = 1

Atom	X	Y	Z	Atom	X	Y	Z
Si	-2.57388	-0.0031	0.007568	H	-3.7454	-2.59457	-4.14753
N	3.574707	-0.00407	-0.0082	C	-3.27957	-1.50249	-2.35009
C	-3.18197	1.572364	-0.84798	H	-2.98503	-0.61218	-2.90019
C	-3.18568	-0.04546	1.798418	C	0.053459	-0.73917	-0.95385
C	-3.23001	-1.50349	-0.94221	H	-0.47163	-1.30611	-1.71734
C	-0.67881	-0.03704	0.020338	C	1.446946	-0.72403	-0.97614
C	-2.36976	2.716477	-0.94906	H	1.991203	-1.25279	-1.75178
H	-1.35289	2.689865	-0.56702	C	2.155321	-0.01381	0.000517
C	-2.83973	3.890203	-1.54036	C	1.450324	0.682299	0.990388
H	-2.19098	4.75906	-1.60876	H	1.997497	1.211837	1.763476
C	-4.13871	3.946142	-2.04706	C	0.056662	0.671991	0.989574
H	-4.50561	4.858333	-2.50915	H	-0.46477	1.208106	1.778213
C	-4.96265	2.822591	-1.96286	C	4.402715	-1.13378	-0.02566
H	-5.97317	2.856997	-2.36058	C	4.058837	-2.48843	-0.00473
C	-4.48691	1.650689	-1.3727	H	3.022538	-2.80638	0.016071
H	-5.13857	0.781532	-1.33089	C	5.094374	-3.42024	-0.00449
C	-4.30048	0.707146	2.210657	H	4.851599	-4.47872	0.009713
H	-4.80107	1.359505	1.500463	C	6.441321	-3.02007	-0.01886
C	-4.77571	0.640958	3.521415	H	7.224131	-3.77225	-0.02044
H	-5.63692	1.234697	3.815117	C	6.777831	-1.66989	-0.02508
C	-4.14333	-0.18217	4.453993	H	7.819323	-1.36114	-0.02529
H	-4.51092	-0.23342	5.475023	C	5.756872	-0.71245	-0.0248
C	-3.0323	-0.93519	4.070571	C	5.746604	0.735307	-0.00925
H	-2.53115	-1.5737	4.792831	C	6.753611	1.707349	-0.01719
C	-2.55992	-0.86389	2.759202	H	7.799403	1.41363	-0.02592
H	-1.68418	-1.44684	2.484676	C	6.39774	3.052594	-0.01992
C	-3.64753	-2.67034	-0.27648	H	7.169662	3.815928	-0.02461
H	-3.63665	-2.7029	0.809517	C	5.045132	3.433419	-0.02277
C	-4.08718	-3.79073	-0.98342	H	4.787171	4.488336	-0.03456
H	-4.40734	-4.67859	-0.44518	C	4.023214	2.486729	-0.01439
C	-4.12018	-3.76906	-2.3783	H	2.982222	2.789453	-0.02674
H	-4.46351	-4.63977	-2.92989	C	4.386585	1.137146	0.003008
C	-3.71605	-2.62102	-3.06173				

Table S2. Cartesian coordinates for optimized structure for **3PCBP-D$_0$**.

Symbolic Z-matrix:
Charge = 1 Multiplicity = 2

Atom	X	Y	Z	Atom	X	Y	Z
Si	-2.61424	-0.0108	0.011189	H	-3.57179	-3.41252	-3.56728
N	3.543584	-0.00222	-0.0005	C	-3.18667	-1.96921	-2.01586
C	-3.16436	1.372642	-1.14598	H	-2.881	-1.20473	-2.72626
C	-3.18096	0.306312	1.779248	C	0.040992	-0.84122	-0.86406
C	-3.20306	-1.68136	-0.6354	H	-0.48837	-1.47946	-1.56493
C	-0.69242	-0.03152	0.028943	C	1.429262	-0.8455	-0.88062
C	-2.3976	2.538988	-1.32921	H	1.97135	-1.44289	-1.60558
H	-1.43534	2.640199	-0.83348	C	2.131281	-0.0118	0.009311
C	-2.84656	3.58027	-2.14136	C	1.431499	0.807715	0.913589
H	-2.23752	4.470541	-2.26871	H	1.975778	1.406662	1.635704
C	-4.07772	3.4773	-2.79094	C	0.042482	0.779139	0.920831
H	-4.42889	4.286527	-3.42415	H	-0.48395	1.381853	1.654621
C	-4.85505	2.32989	-2.62572	C	4.366651	-1.13682	-0.00515
H	-5.81349	2.244233	-3.12899	C	4.003448	-2.4871	0.073475
C	-4.40107	1.288665	-1.81485	H	2.967115	-2.79517	0.13726
H	-5.01628	0.399607	-1.7069	C	5.034203	-3.42769	0.086069
C	-4.20255	1.235226	2.050307	H	4.789511	-4.48277	0.138935
H	-4.64894	1.798598	1.235848	C	6.375535	-3.02801	0.046938
C	-4.65616	1.449802	3.35292	H	7.157269	-3.78009	0.06331
H	-5.44659	2.171096	3.537834	C	6.730652	-1.66731	0.007146
C	-4.09514	0.739896	4.414942	H	7.775617	-1.3754	0.004955
H	-4.44803	0.906041	5.428377	C	5.721898	-0.71733	-0.01604
C	-3.07729	-0.1845	4.171372	C	5.711804	0.743192	-0.02005
H	-2.63704	-0.73913	4.994961	C	6.706871	1.707097	-0.05918
C	-2.62613	-0.39607	2.868365	H	7.755669	1.429656	-0.07388
H	-1.83002	-1.11887	2.702513	C	6.332403	3.062673	-0.09284
C	-3.64583	-2.691	0.239709	H	7.103244	3.825545	-0.12158
H	-3.69128	-2.4984	1.307699	C	4.985208	3.443668	-0.1101
C	-4.048	-3.9379	-0.2411	H	4.725154	4.495288	-0.15855
H	-4.39362	-4.69873	0.452502	C	3.967995	2.48875	-0.08125
C	-4.01566	-4.2035	-1.61076	H	2.926492	2.782014	-0.12804
H	-4.33251	-5.17217	-1.98566	C	4.351055	1.143854	-0.00908
C	-3.58577	-3.21571	-2.49924				

Table S3. Cartesian coordinates for optimized structure for **3PCBP-T$_0$**.

Symbolic Z-matrix:
Charge = 0 Multiplicity = 3

Atom	X	Y	Z	Atom	X	Y	Z
Si	-2.58611	-0.00272	0.007499	H	-3.75377	-2.75556	-4.04397
N	3.56117	0.003207	-0.01316	C	-3.28945	-1.59373	-2.29039
C	-3.19724	1.538245	-0.90722	H	-2.99129	-0.72686	-2.87492
C	-3.19851	0.022752	1.798751	C	0.041333	-0.77787	-0.9221
C	-3.24308	-1.5386	-0.88351	H	-0.48319	-1.37804	-1.6602
C	-0.69194	-0.03484	0.02067	C	1.4345	-0.76346	-0.9452
C	-2.38652	2.678684	-1.05237	H	1.978432	-1.3275	-1.69546
H	-1.36933	2.667764	-0.67041	C	2.143231	-0.00849	-0.00224
C	-2.85833	3.828456	-1.68762	C	1.437136	0.730168	0.956206
H	-2.21069	4.694872	-1.78949	H	1.983453	1.295872	1.703547
C	-4.15767	3.86348	-2.19531	C	0.043867	0.716481	0.957529
H	-4.52602	4.756991	-2.69152	H	-0.47752	1.285395	1.72296
C	-4.98007	2.742872	-2.068	C	4.38912	-1.13939	-0.02702
H	-5.99082	2.760771	-2.46627	C	4.066671	-2.46292	0.000863
C	-4.50247	1.594891	-1.43391	H	3.039746	-2.80599	0.028552
H	-5.15294	0.727063	-1.35877	C	5.156453	-3.42244	0.00639
C	-4.31877	0.783329	2.180493	H	4.917271	-4.48028	0.026845
H	-4.82344	1.403584	1.44487	C	6.498649	-3.00833	-0.01547
C	-4.79432	0.765913	3.492673	H	7.282278	-3.76052	-0.01769
H	-5.65984	1.364803	3.76231	C	6.839368	-1.66681	-0.03512
C	-4.15673	-0.01506	4.457433	H	7.874471	-1.3445	-0.05132
H	-4.52456	-0.02829	5.479589	C	5.770424	-0.67769	-0.02927
C	-3.04023	-0.77485	4.10453	C	5.763824	0.696892	-0.00738
H	-2.53501	-1.38063	4.851736	C	6.814944	1.698487	-0.00376
C	-2.56764	-0.75243	2.791461	H	7.8548	1.391065	0.005307
H	-1.68774	-1.33962	2.540368	C	6.4565	3.026064	-0.01701
C	-3.66508	-2.67696	-0.1728	H	7.2281	3.790771	-0.0171
H	-3.65655	-2.66622	0.913655	C	5.096747	3.430208	-0.03212
C	-4.10599	-3.82361	-0.83548	H	4.853753	4.487202	-0.0513
H	-4.42964	-4.68849	-0.26299	C	4.032698	2.474027	-0.02325
C	-4.13573	-3.85758	-2.23019	H	3.000337	2.801883	-0.04232
H	-4.47998	-4.74878	-2.74743	C	4.367541	1.14596	-0.00097
C	-3.72705	-2.73872	-2.9579				

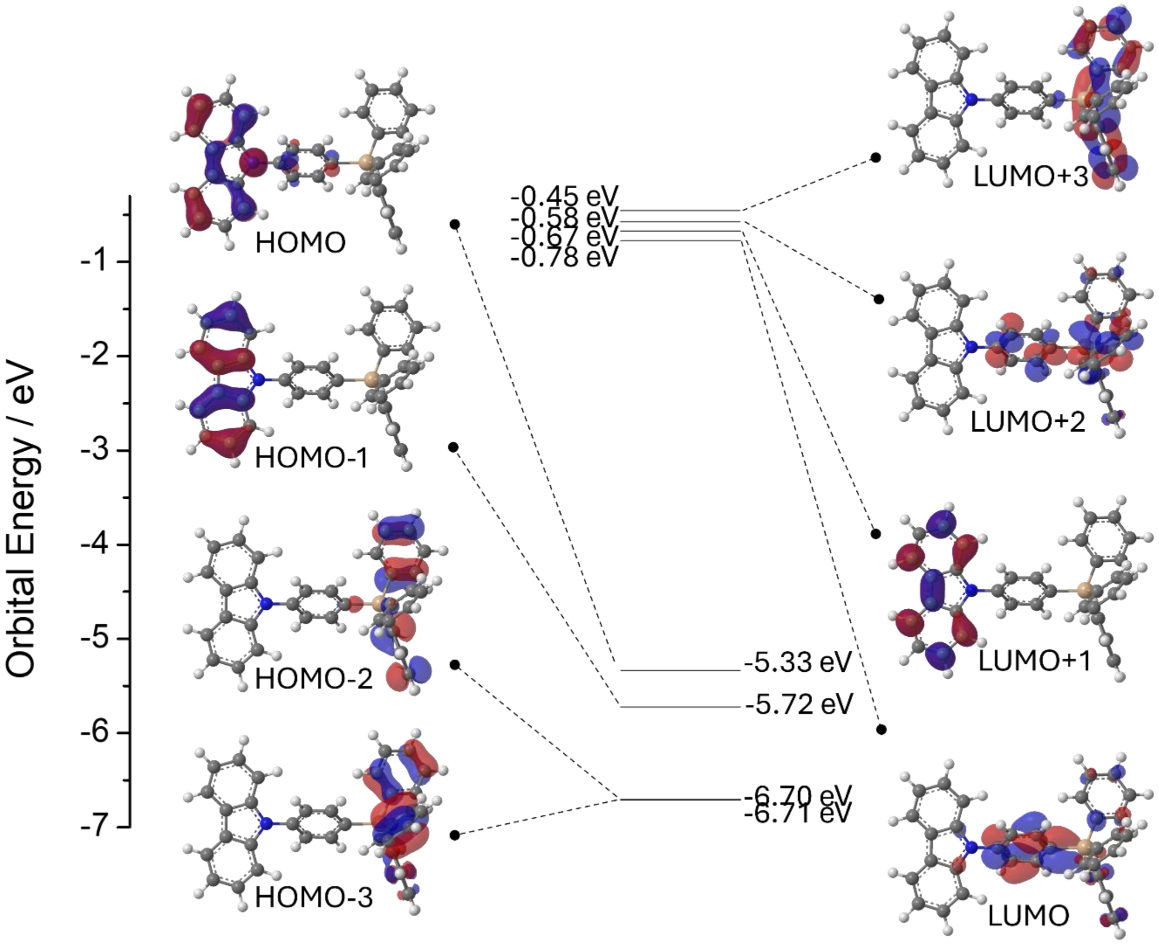

Figure S1. Frontier orbitals and orbital energy levels (isodensity contour = 0.04 a.u.) for selected occupied and unoccupied molecular orbitals of **3PCBP-S$_0$**.

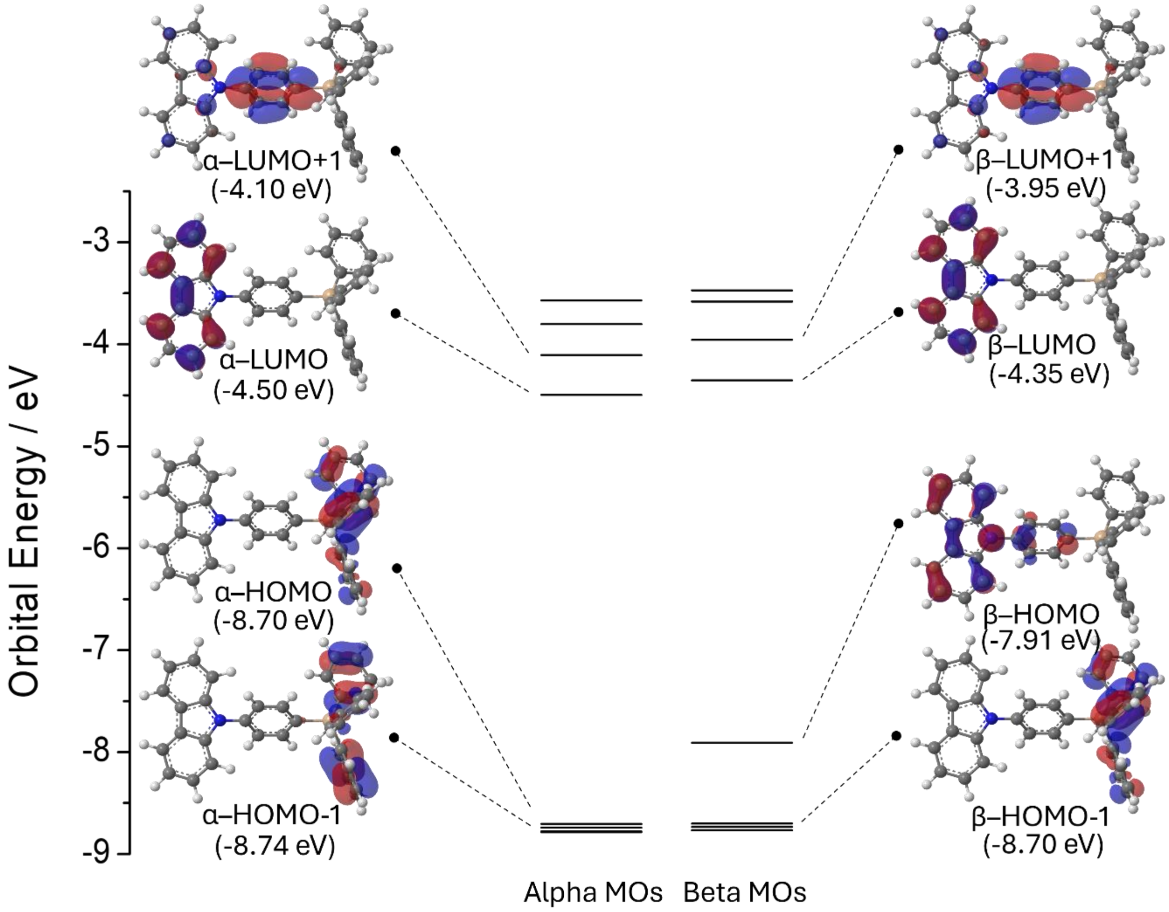

Figure S2. Frontier orbitals and orbital energy levels (isodensity contour = 0.04 a.u.) for selected occupied and unoccupied molecular orbitals of **3PCBP-D$_0$**.

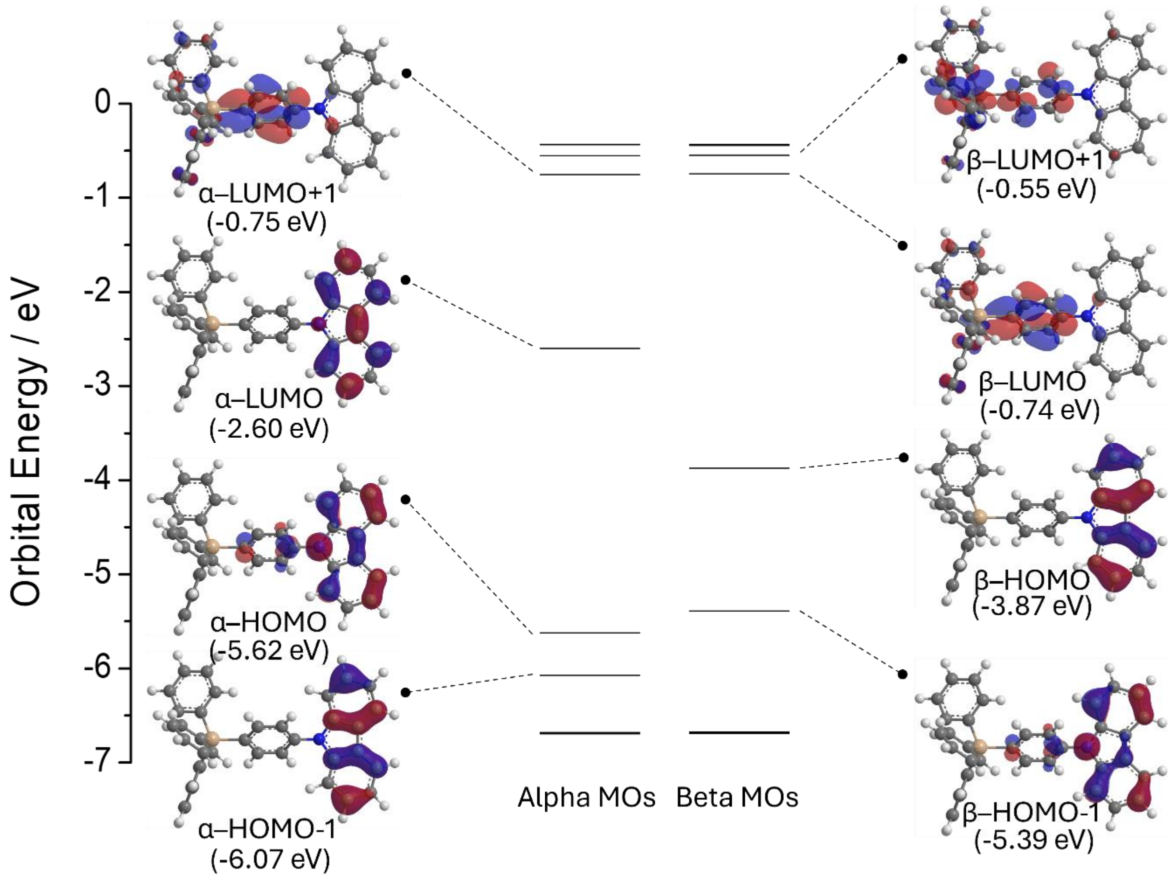

Figure S3. Frontier orbitals and orbital energy levels (isodensity contour = 0.04 a.u.) for selected occupied and unoccupied molecular orbitals of **3PCBP-T$_0$**.

Table S4. Cartesian coordinates for optimized structure for **2MCBP-S$_0$**.

Symbolic Z-matrix:
Charge = 0 Multiplicity = 1

Atom	X	Y	Z	Atom	X	Y	Z
Si	-0.00039	3.15639	-0.58878	H	-8.25423	-1.27725	4.06054
N	-5.03039	-0.32405	0.033798	C	-6.48938	-0.1817	3.473372
N	5.030646	-0.31332	0.084729	H	-6.36175	0.269622	4.452911
C	0.041312	3.951765	-2.305	C	-5.58085	0.125463	2.462993
H	-0.81825	4.610941	-2.46899	H	-4.75932	0.810226	2.640937
H	0.046842	3.199966	-3.10071	C	-5.7702	-0.47275	1.214037
H	0.945994	4.558551	-2.41771	C	2.654717	2.532149	0.381552
C	-0.04254	4.515383	0.727051	H	2.638872	3.515377	0.844188
H	0.816138	5.190479	0.642518	C	3.807219	1.759431	0.517482
H	-0.04605	4.099423	1.73965	H	4.666899	2.141297	1.058356
H	-0.948	5.12121	0.615468	C	3.865912	0.484838	-0.05801
C	-1.53737	2.060883	-0.43089	C	2.761989	0.002897	-0.77324
C	1.537164	2.078142	-0.34152	H	2.796213	-0.9945	-1.19921
C	-1.63686	1.091773	0.585636	C	1.62509	0.795204	-0.91464
H	-0.80002	0.929131	1.260391	H	0.778992	0.390471	-1.46442
C	-2.77342	0.30144	0.739561	C	5.652574	-0.64864	1.294316
H	-2.8164	-0.45922	1.512196	C	5.281776	-0.32851	2.603373
C	-3.86563	0.471748	-0.12113	H	4.404231	0.274098	2.80886
C	-3.7952	1.431818	-1.13719	C	6.074485	-0.81664	3.639732
H	-4.64584	1.576513	-1.79511	H	5.806979	-0.57974	4.665312
C	-2.64289	2.202041	-1.28854	C	7.206651	-1.60933	3.386172
H	-2.61774	2.935666	-2.08986	H	7.804184	-1.97196	4.216822
C	-5.63557	-1.09643	-0.96598	C	7.562706	-1.93699	2.081503
C	-5.24487	-1.29762	-2.2928	H	8.431727	-2.55898	1.886815
H	-4.36278	-0.81596	-2.69947	C	6.783057	-1.46024	1.021398
C	-6.02371	-2.14592	-3.0767	C	6.848951	-1.61971	-0.41622
H	-5.7406	-2.31652	-4.11123	C	7.732462	-2.28572	-1.27344
C	-7.16163	-2.78579	-2.55694	H	8.575153	-2.83987	-0.87
H	-7.74795	-3.43917	-3.19557	C	7.520587	-2.22357	-2.64731
C	-7.53763	-2.59307	-1.23113	H	8.198027	-2.73717	-3.32245
H	-8.41121	-3.0966	-0.82703	C	6.438824	-1.49543	-3.17089
C	-6.7722	-1.74634	-0.42112	H	6.295575	-1.4507	-4.24651
C	-6.85956	-1.34661	0.967796	C	5.544294	-0.82405	-2.3404
C	-7.75744	-1.63859	2.001012	H	4.718222	-0.25629	-2.7536
H	-8.59578	-2.30699	1.826665	C	5.753603	-0.90224	-0.96068
C	-7.56551	-1.05742	3.250682				

Table S5. Cartesian coordinates for optimized structure for **2MCBP-D$_0$**.

Symbolic Z-matrix:
Charge = 1 Multiplicity = 2

Atom	X	Y	Z	Atom	X	Y	Z
Si	-0.00056	3.263832	-0.01291	H	-9.07747	-0.88614	3.062704
N	-4.98024	-0.33822	-0.14901	C	-7.23985	0.202502	2.778222
N	4.980275	-0.33545	0.151716	H	-7.3677	0.788701	3.682133
C	0.076815	4.32878	-1.56658	C	-6.11394	0.413551	1.983455
H	-0.81238	4.961354	-1.65946	H	-5.37634	1.16138	2.249274
H	0.163509	3.735859	-2.48292	C	-5.98367	-0.36487	0.827962
H	0.94759	4.991098	-1.5237	C	2.372993	2.097536	1.187624
C	-0.07807	4.341675	1.531821	H	2.156781	2.719719	2.051063
H	0.810488	4.975984	1.618775	C	3.505256	1.287428	1.228831
H	-0.16345	3.756425	2.453189	H	4.158697	1.29681	2.094621
H	-0.94954	5.002733	1.483921	C	3.826554	0.488688	0.123056
C	-1.53556	2.144532	-0.07498	C	3.006401	0.509105	-1.01466
C	1.534645	2.145309	0.058341	H	3.239438	-0.13156	-1.85836
C	-1.88676	1.340821	1.027767	C	1.882061	1.327575	-1.03527
H	-1.26612	1.341615	1.920117	H	1.258085	1.31666	-1.92523
C	-3.01083	0.521832	1.013585	C	5.311395	-1.24003	1.167295
H	-3.24681	-0.10797	1.864613	C	4.592597	-1.57813	2.319539
C	-3.82684	0.486581	-0.12674	H	3.638885	-1.11719	2.547426
C	-3.50178	1.27122	-1.24147	C	5.1435	-2.54232	3.16243
H	-4.15213	1.269365	-2.10963	H	4.612325	-2.82246	4.066012
C	-2.36988	2.082138	-1.20654	C	6.3653	-3.15991	2.858625
H	-2.15081	2.69345	-2.07701	H	6.768895	-3.9075	3.533414
C	-5.30725	-1.25611	-1.15391	C	7.066268	-2.8339	1.691235
C	-4.58402	-1.60901	-2.29892	H	8.002576	-3.33054	1.457421
H	-3.6296	-1.15074	-2.52923	C	6.538175	-1.87104	0.836293
C	-5.13145	-2.58435	-3.13118	C	6.96636	-1.31629	-0.43849
H	-4.59679	-2.87611	-4.02901	C	8.080792	-1.51719	-1.24782
C	-6.3542	-3.19834	-2.82391	H	8.849205	-2.23067	-0.96766
H	-6.75502	-3.95484	-3.49037	C	8.20172	-0.77502	-2.42878
C	-7.05967	-2.85731	-1.66355	H	9.065676	-0.92404	-3.06789
H	-7.99671	-3.35117	-1.42679	C	7.22877	0.167692	-2.79076
C	-6.53509	-1.88316	-0.8193	H	7.353047	0.742098	-3.7027
C	-6.96824	-1.31193	0.446492	C	6.105758	0.388764	-1.99461
C	-8.08563	-1.50263	1.254206	H	5.366932	1.132862	-2.2674
H	-8.85278	-2.21998	0.980519	C	5.980057	-0.37456	-0.82859
C	-8.21118	-0.74517	2.42493				

Table S6. Cartesian coordinates for optimized structure for **2MCBP-T$_0$**.

Symbolic Z-matrix:
Charge = 0 Multiplicity = 3

Atom	X	Y	Z	Atom	X	Y	Z
Si	0.01965	3.181384	0.440031	H	-8.20107	-2.65589	3.448072
N	-5.02307	-0.29794	-0.0619	C	-6.44966	-1.41702	3.249024
N	5.044268	-0.33165	-0.02987	H	-6.29695	-1.34073	4.320247
C	0.064469	4.479192	-0.93618	C	-5.521	-0.74362	2.364758
H	-0.7924	5.159694	-0.88136	H	-4.69949	-0.16825	2.773741
H	0.066014	4.017898	-1.92899	C	-5.73109	-0.85375	1.022244
H	0.971669	5.087018	-0.85279	C	2.677775	2.28224	1.152295
C	-0.01547	4.055771	2.117765	H	2.667379	3.07302	1.897679
H	0.846375	4.719235	2.249551	C	3.828935	1.504683	1.033862
H	-0.02111	3.341358	2.947246	H	4.69287	1.698857	1.661037
H	-0.91811	4.669914	2.2044	C	3.880596	0.471956	0.090438
C	-1.52042	2.097867	0.245377	C	2.770976	0.236737	-0.7316
C	1.554612	2.076367	0.331269	H	2.799803	-0.57908	-1.44649
C	-1.62188	0.854169	0.897876	C	1.635444	1.034754	-0.61245
H	-0.78346	0.480289	1.480478	H	0.784707	0.821518	-1.25483
C	-2.762	0.060923	0.797373	C	5.672667	-1.02686	1.011236
H	-2.80664	-0.90551	1.28827	C	5.309961	-1.13051	2.357101
C	-3.85652	0.500997	0.040772	H	4.434536	-0.62212	2.745217
C	-3.78384	1.735955	-0.61507	C	6.107885	-1.91689	3.185116
H	-4.63458	2.086355	-1.18988	H	5.846701	-2.01128	4.235064
C	-2.62889	2.51008	-0.5159	C	7.237338	-2.59082	2.690495
H	-2.60345	3.46197	-1.03968	H	7.839065	-3.19401	3.36327
C	-5.66217	-0.66181	-1.25461	C	7.585384	-2.49562	1.346569
C	-5.33357	-0.4005	-2.55664	H	8.452353	-3.02571	0.96268
H	-4.46113	0.185287	-2.81977	C	6.800363	-1.71266	0.492352
C	-6.1799	-0.94048	-3.58122	C	6.857706	-1.41622	-0.92373
H	-5.9356	-0.74601	-4.62004	C	7.735214	-1.78162	-1.9511
C	-7.3211	-1.71793	-3.2653	H	8.579419	-2.43357	-1.74548
H	-7.93517	-2.09838	-4.07659	C	7.515492	-1.2945	-3.23591
C	-7.66394	-1.99936	-1.96242	H	8.188227	-1.57191	-4.04152
H	-8.53641	-2.59622	-1.72084	C	6.431783	-0.43988	-3.50005
C	-6.82319	-1.4811	-0.8966	H	6.282347	-0.06227	-4.50731
C	-6.85744	-1.59961	0.47213	C	5.543099	-0.06104	-2.49637
C	-7.76463	-2.27425	1.389638	H	4.715476	0.606873	-2.7071
H	-8.5997	-2.84642	1.001198	C	5.760318	-0.56514	-1.21095
C	-7.53416	-2.16123	2.747806				

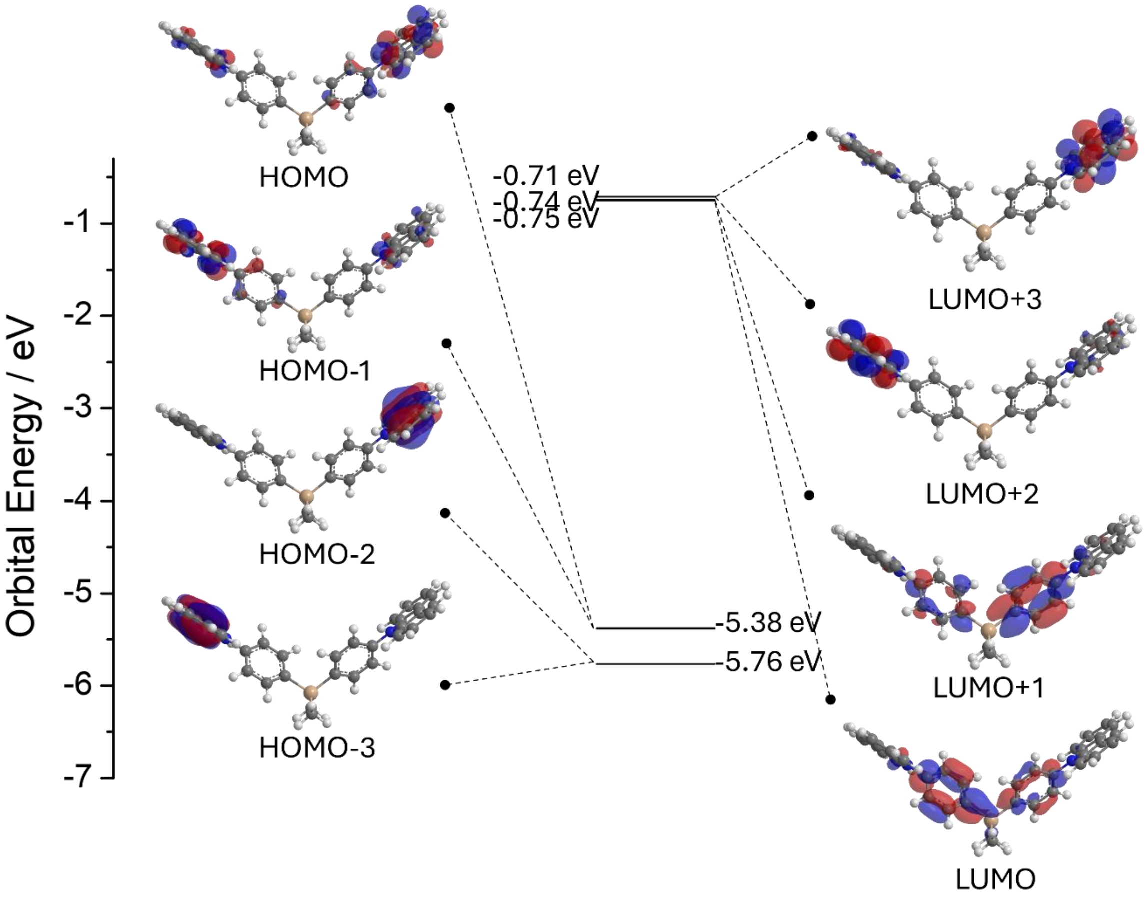

Figure S4. Frontier orbitals and orbital energy levels (isodensity contour = 0.04 a.u.) for selected occupied and unoccupied molecular orbitals of **2MCBP-S$_0$**.

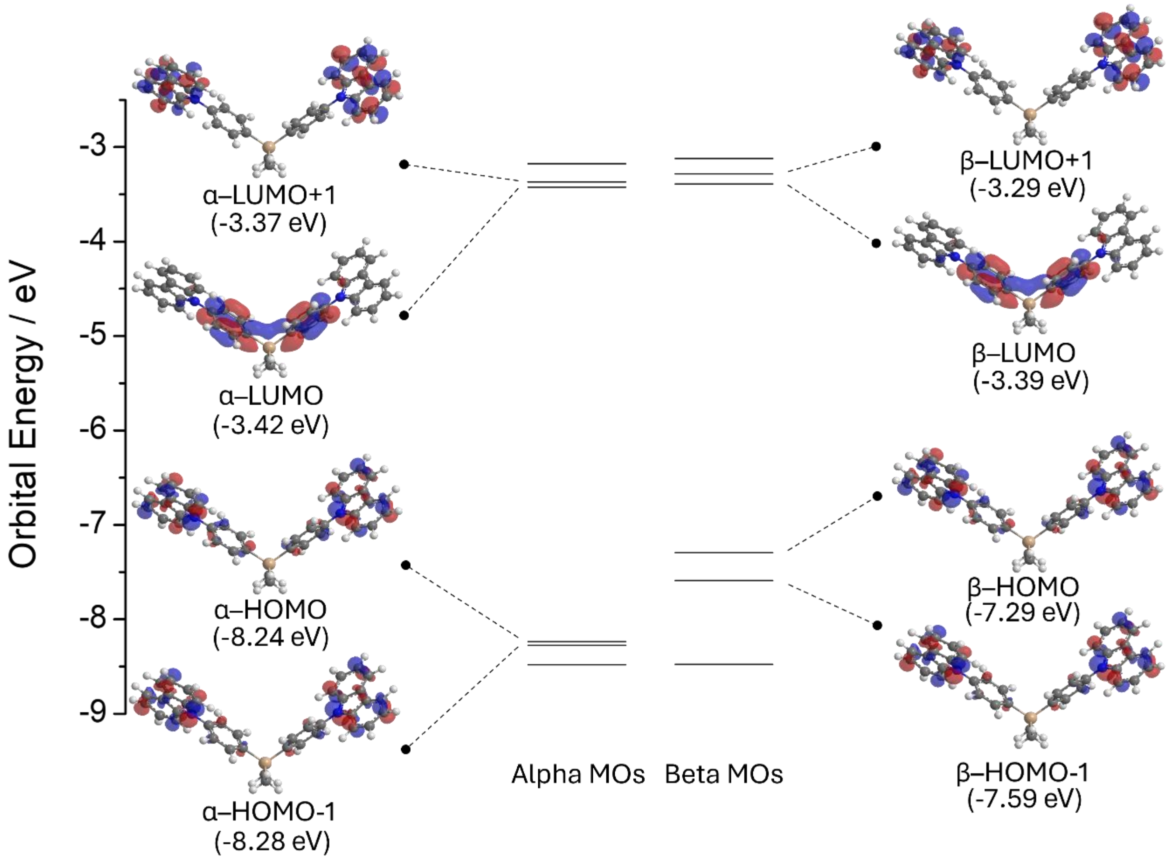

Figure S5. Frontier orbitals and orbital energy levels (isodensity contour = 0.04 a.u.) for selected occupied and unoccupied molecular orbitals of **2MCBP-D$_0$**.

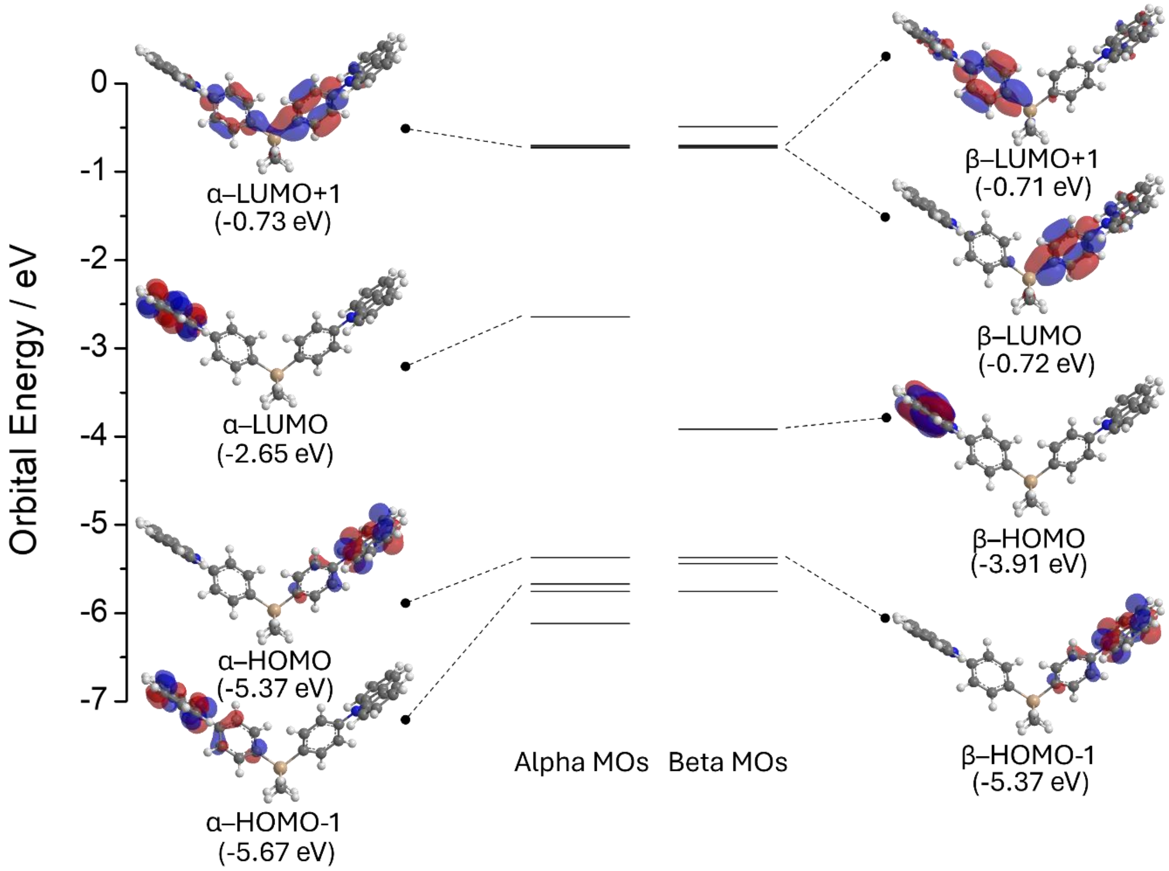

Figure S6. Frontier orbitals and orbital energy levels (isodensity contour = 0.04 a.u.) for selected occupied and unoccupied molecular orbitals of **2MCBP-T$_0$**.

Table S7. Cartesian coordinates for optimized structure for **MCBP-S$_0$**.

Symbolic Z-matrix:
Charge = 0 Multiplicity = 1

Atom	X	Y	Z	Atom	X	Y	Z
Si	0.040264	0.007595	-1.9634	C	-4.23508	5.078642	0.439976
N	-2.90529	-5.01556	0.001275	C	-5.17779	4.051166	0.535458
N	-2.89619	5.02494	0.030119	H	-4.93022	3.031742	0.261736
N	5.796598	-0.01204	0.189337	C	-6.4487	4.378284	1.003064
C	0.081112	0.017162	-3.8539	H	-7.19844	3.596782	1.084312
H	0.560365	-0.88571	-4.24601	C	-6.7776	5.693001	1.373904
H	0.63953	0.87935	-4.23266	H	-7.77799	5.91454	1.732551
H	-0.92889	0.065772	-4.27356	C	-5.83045	6.709065	1.290966
C	-0.84797	1.559992	-1.3443	H	-6.08203	7.722997	1.588592
C	1.810702	-0.01428	-1.2947	C	-4.54562	6.407569	0.824971
C	-0.8825	-1.52981	-1.35819	C	-3.34335	7.192157	0.636283
C	-1.82667	-1.47071	-0.31753	C	-3.03547	8.544586	0.823549
H	-2.0486	-0.5174	0.153771	H	-3.7888	9.231934	1.197688
C	-2.48571	-2.61052	0.141103	C	-1.75542	8.997096	0.518688
H	-3.19022	-2.54572	0.963754	H	-1.50379	10.04346	0.661157
C	-2.23021	-3.85303	-0.45102	C	-0.78462	8.111481	0.021167
C	-1.30173	-3.93729	-1.4972	H	0.206083	8.485268	-0.2201
H	-1.11856	-4.89521	-1.97265	C	-1.06438	6.760745	-0.17392
C	-0.63714	-2.79275	-1.93078	H	-0.31177	6.087121	-0.56785
H	0.0777	-2.89178	-2.74404	C	-2.34718	6.308982	0.148229
C	-4.29353	-5.16343	0.123019	C	2.215406	-0.89911	-0.27942
C	-5.31479	-4.26249	-0.19129	H	1.502504	-1.60869	0.130825
H	-5.0951	-3.27491	-0.58092	C	3.522412	-0.9106	0.205992
C	-6.62976	-4.6784	0.00545	H	3.819964	-1.62166	0.969618
H	-7.4402	-3.99488	-0.23001	C	4.467214	-0.01299	-0.30506
C	-6.92763	-5.96053	0.497043	C	4.086911	0.885733	-1.31085
H	-7.96332	-6.25228	0.640988	H	4.812108	1.59563	-1.69475
C	-5.90713	-6.85868	0.79398	C	2.782532	0.871833	-1.79853
H	-6.13841	-7.85367	1.163347	H	2.519126	1.58157	-2.57915
C	-4.57696	-6.46657	0.604805	C	6.171142	0.090315	1.535951
C	-3.30495	-7.13438	0.785589	C	5.372032	0.246567	2.671939
C	-2.93993	-8.4057	1.243155	H	4.291309	0.292792	2.599161
H	-3.70135	-9.12157	1.539299	C	6.011322	0.348586	3.905531
C	-1.59068	-8.7374	1.319509	H	5.410993	0.46854	4.80254
H	-1.2949	-9.7208	1.671487	C	7.411628	0.3029	4.011786
C	-0.60463	-7.80695	0.950503	H	7.878024	0.383006	4.988785
H	0.44356	-8.08073	1.026387	C	8.202775	0.162153	2.875772
C	-0.94053	-6.53525	0.491319	H	9.285678	0.137997	2.957474
H	-0.17216	-5.82054	0.218854	C	7.586004	0.058117	1.623705
C	-2.29763	-6.21294	0.402395	C	8.088667	-0.0732	0.27214
C	-0.33011	2.353989	-0.3056	C	9.373819	-0.17315	-0.27304
H	0.612931	2.082347	0.160095	H	10.24712	-0.14453	0.372283
C	-1.00116	3.484927	0.157944	C	9.517241	-0.31583	-1.64979
H	-0.59611	4.067589	0.978746	H	10.50885	-0.39297	-2.08482

C	-2.21363	3.868583	-0.42689	C	8.387642	-0.36779	-2.48375
C	-2.74628	3.100016	-1.47072	H	8.51939	-0.48987	-3.55483
H	-3.67468	3.407809	-1.94038	C	7.09745	-0.27001	-1.96728
C	-2.07372	1.962179	-1.90927	H	6.231583	-0.32229	-2.61764
H	-2.51356	1.386838	-2.7204	C	6.96005	-0.11132	-0.58543

Table S8. Cartesian coordinates for optimized structure for **MCBP-D$_0$**.

Symbolic Z-matrix:
Charge = 1 Multiplicity = 2

Atom	X	Y	Z	Atom	X	Y	Z
Si	-0.02612	0.029886	-2.00572	C	4.973062	-4.46274	0.125785
N	2.120506	5.380076	0.111785	C	5.826067	-3.36364	-0.01732
N	3.587662	-4.54333	-0.06952	H	5.454372	-2.39594	-0.33322
N	-5.70032	-0.83619	0.181685	C	7.176766	-3.55315	0.270585
C	-0.06582	0.050986	-3.89182	H	7.86287	-2.71905	0.164285
H	-0.74731	0.820189	-4.26832	C	7.662561	-4.79755	0.699085
H	-0.39972	-0.91061	-4.29422	H	8.71956	-4.91405	0.914785
H	0.925183	0.255981	-4.30912	C	6.800756	-5.88565	0.859785
C	1.079376	-1.38572	-1.40222	H	7.181752	-6.84145	1.205885
C	-1.77692	-0.22161	-1.32662	C	5.445657	-5.72174	0.575785
C	0.65839	1.672582	-1.35612	C	4.292953	-6.60243	0.651285
C	1.44739	1.739679	-0.19262	C	4.128147	-7.94233	0.999685
H	1.692686	0.828878	0.346985	H	4.978344	-8.54194	1.309585
C	1.925395	2.951777	0.300485	C	2.851244	-8.50633	0.935485
H	2.505495	2.984474	1.216585	H	2.71084	-9.54793	1.204985
C	1.633901	4.142278	-0.37872	C	1.749648	-7.74572	0.515485
C	0.86	4.103682	-1.54682	H	0.769946	-8.20902	0.459285
H	0.660505	5.021282	-2.08972	C	1.889054	-6.40502	0.160185
C	0.380695	2.884384	-2.01752	H	1.037756	-5.82802	-0.18112
H	-0.20971	2.884486	-2.92972	C	3.166356	-5.84153	0.247885
C	3.452207	5.66167	0.443285	C	-2.21152	0.397595	-0.13952
C	4.584704	4.848665	0.329185	H	-1.54361	1.065592	0.396985
H	4.518199	3.835565	-0.04972	C	-3.49632	0.202901	0.362185
C	5.810506	5.39296	0.708985	H	-3.82032	0.716002	1.261185
H	6.706103	4.784856	0.633285	C	-4.39102	-0.6323	-0.32042
C	5.907912	6.711559	1.178485	C	-3.98442	-1.2625	-1.50522
H	6.876414	7.106955	1.466185	H	-4.66753	-1.92879	-2.02092
C	4.775116	7.524664	1.269485	C	-2.69912	-1.0506	-1.99502
H	4.86112	8.548964	1.618485	H	-2.41193	-1.5552	-2.91352
C	3.537113	7.00247	0.897485	C	-6.02472	-1.21309	1.491985
C	2.194116	7.553076	0.839385	C	-5.17813	-1.51169	2.564585
C	1.648321	8.792978	1.168785	H	-4.10022	-1.4591	2.466585
H	2.278525	9.592075	1.546485	C	-5.76993	-1.89199	3.768085
C	0.273922	8.990784	1.012185	H	-5.13743	-2.12609	4.618285
H	-0.16167	9.952286	1.263385	C	-7.16383	-1.98338	3.897485
C	-0.55208	7.958388	0.542785	H	-7.59483	-2.28188	4.847485
H	-1.61848	8.131193	0.440685	C	-8.00263	-1.70428	2.815485
C	-0.03049	6.709586	0.208885	H	-9.07973	-1.79247	2.918585
H	-0.67589	5.911388	-0.13882	C	-7.43452	-1.31958	1.601685
C	1.349011	6.525579	0.348585	C	-7.98532	-0.98858	0.298985
C	0.773273	-2.12602	-0.24502	C	-9.28392	-0.89897	-0.20062
H	-0.12353	-1.89371	0.322685	H	-10.137	-1.11637	0.434385
C	1.594568	-3.15732	0.204685	C	-9.47232	-0.51647	-1.53122
H	1.355666	-3.69552	1.115685	H	-10.4782	-0.44497	-1.93162

C	2.751967	-3.48813	-0.51282	C	-8.37692	-0.21308	-2.35372
C	3.07697	-2.77273	-1.67402	H	-8.54782	0.095223	-3.38002
H	3.955669	-3.04893	-2.24692	C	-7.06962	-0.29298	-1.87692
C	2.250374	-1.73763	-2.10132	H	-6.22922	-0.03999	-2.51262
H	2.521977	-1.20823	-3.01062	C	-6.88762	-0.69818	-0.55042

Table S9. Cartesian coordinates for optimized structure for **MCBP-T$_0$**.

Symbolic Z-matrix:
Charge = 0 Multiplicity = 3

Atom	X	Y	Z	Atom	X	Y	Z
Si	0.043642	0.001066	-1.96823	C	-4.6507	4.70854	0.381728
N	-2.47521	-5.24189	0.016843	C	-5.51276	3.650879	0.442821
N	-3.29602	4.762793	0.013581	H	-5.20853	2.646555	0.174235
N	5.783072	0.453074	0.183611	C	-6.85635	3.905609	0.893506
C	0.083283	0.007431	-3.85892	H	-7.55508	3.07781	0.949524
H	0.634353	-0.85439	-4.24907	C	-7.27852	5.203185	1.261242
H	0.569215	0.91131	-4.24043	H	-8.30444	5.347531	1.587658
H	-0.92759	-0.02738	-4.27787	C	-6.42031	6.28052	1.214571
C	-0.96775	1.476181	-1.35257	H	-6.74279	7.276185	1.498023
C	1.810736	0.124186	-1.3008	C	-5.051	6.059023	0.777199
C	-0.74904	-1.60587	-1.35819	C	-3.95888	6.88328	0.653262
C	-1.68438	-1.62241	-0.30801	C	-3.75314	8.304121	0.890055
H	-1.9766	-0.68979	0.166383	H	-4.56788	8.912855	1.265958
C	-2.24608	-2.8112	0.155821	C	-2.51243	8.843663	0.621622
H	-2.94514	-2.80304	0.985603	H	-2.33959	9.902765	0.789756
C	-1.89894	-4.02914	-0.44067	C	-1.45383	8.049658	0.130859
C	-0.97751	-4.03894	-1.49631	H	-0.49159	8.505177	-0.07691
H	-0.72382	-4.97909	-1.97486	C	-1.61765	6.635331	-0.10311
C	-0.41013	-2.845	-1.93489	H	-0.7933	6.047854	-0.48853
H	0.302063	-2.88648	-2.75539	C	-2.83664	6.083825	0.165456
C	-3.84604	-5.49963	0.151427	C	2.283351	-0.71765	-0.27833
C	-4.93858	-4.68252	-0.1522	H	1.628345	-1.47794	0.137568
H	-4.80169	-3.68045	-0.54274	C	3.586824	-0.62205	0.207311
C	-6.21443	-5.20161	0.056577	H	3.939019	-1.30106	0.976857
H	-7.07879	-4.58452	-0.17047	C	4.458181	0.343014	-0.31113
C	-6.40488	-6.50349	0.54964	C	4.009055	1.200454	-1.32446
H	-7.41272	-6.8766	0.703077	H	4.676431	1.961986	-1.71427
C	-5.3135	-7.31784	0.835981	C	2.710061	1.079844	-1.81196
H	-5.46151	-8.32814	1.206549	H	2.391979	1.760181	-2.59834
C	-4.02053	-6.82129	0.634603	C	6.146892	0.599598	1.528972
C	-2.69786	-7.38595	0.802779	C	5.336602	0.705499	2.662854
C	-2.22865	-8.62439	1.2555	H	4.255682	0.66601	2.589077
H	-2.92795	-9.3986	1.558039	C	5.964446	0.871125	3.895408
C	-0.85666	-8.84789	1.318696	H	5.355491	0.953752	4.790823
H	-0.48033	-9.8048	1.666792	C	7.363922	0.93643	4.002636
C	0.048798	-7.84193	0.941273	H	7.821426	1.063785	4.97883
H	1.116104	-8.03164	1.00685	C	8.165059	0.845194	2.868564
C	-0.39139	-6.60081	0.486623	H	9.246438	0.906745	2.950907
H	0.315117	-5.8273	0.207437	C	7.559868	0.679103	1.617529
C	-1.77061	-6.38723	0.41104	C	8.072786	0.572181	0.267677
C	-0.51402	2.318751	-0.32199	C	9.362387	0.566806	-0.27618
H	0.450996	2.131264	0.14028	H	10.23014	0.67083	0.368905
C	-1.27502	3.392452	0.1378	C	9.51791	0.420232	-1.6512
H	-0.91441	4.01408	0.950396	H	10.51297	0.415865	-2.08518

C	-2.52052	3.667358	-0.44019	C	8.396664	0.270866	-2.48472
C	-2.99189	2.847702	-1.47538	H	8.538676	0.147468	-3.55433
H	-3.94689	3.069894	-1.93943	C	7.102248	0.273396	-1.96959
C	-2.22671	1.769743	-1.91198	H	6.243682	0.146527	-2.61938
H	-2.62089	1.15425	-2.7172	C	6.951488	0.436274	-0.58963

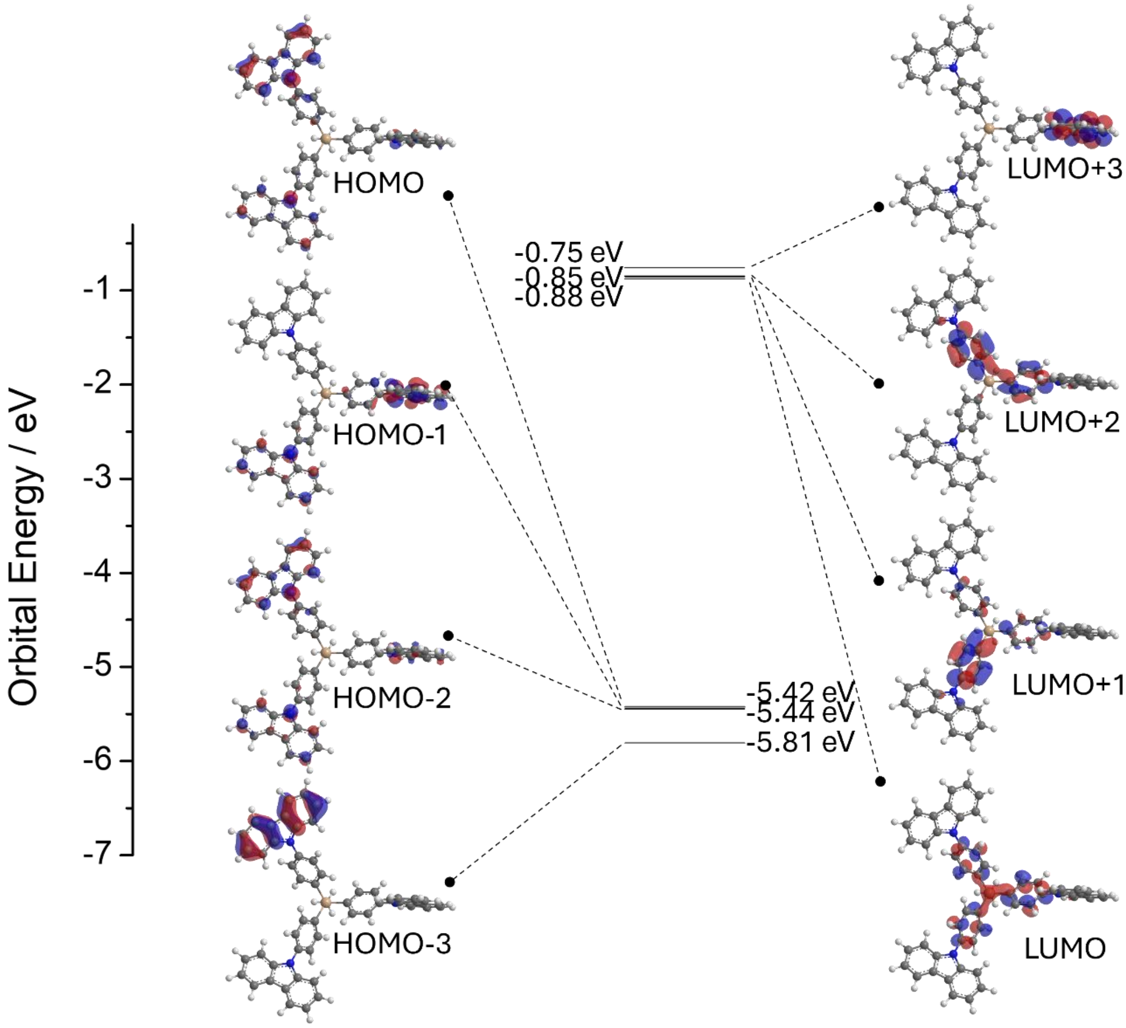

Figure S7. Frontier orbitals and orbital energy levels (isodensity contour = 0.04 a.u.) for selected occupied and unoccupied molecular orbitals of **MCBP-S$_0$**.

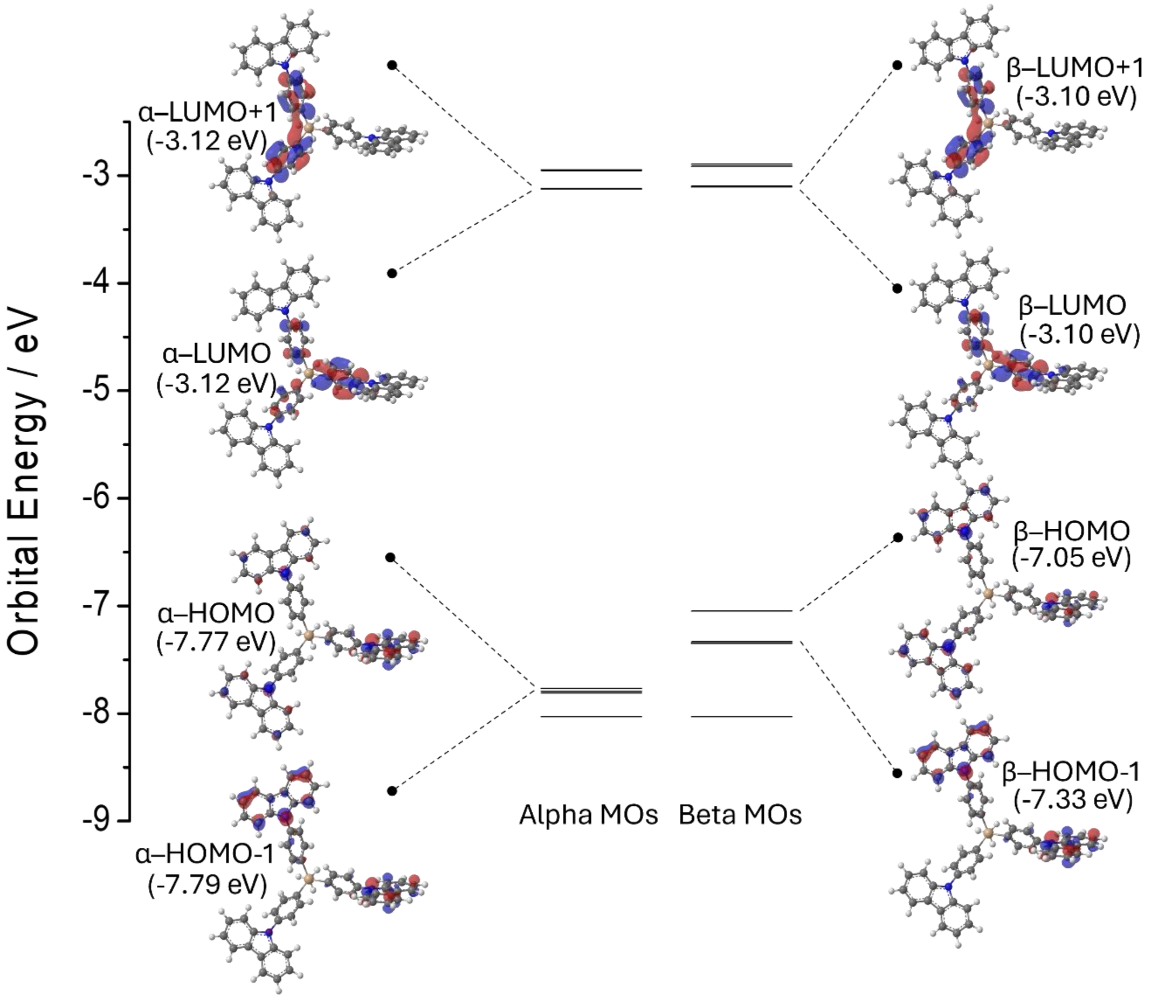

Figure S8. Frontier orbitals and orbital energy levels (isodensity contour = 0.04 a.u.) for selected occupied and unoccupied molecular orbitals of **MCBP-D$_0$**.

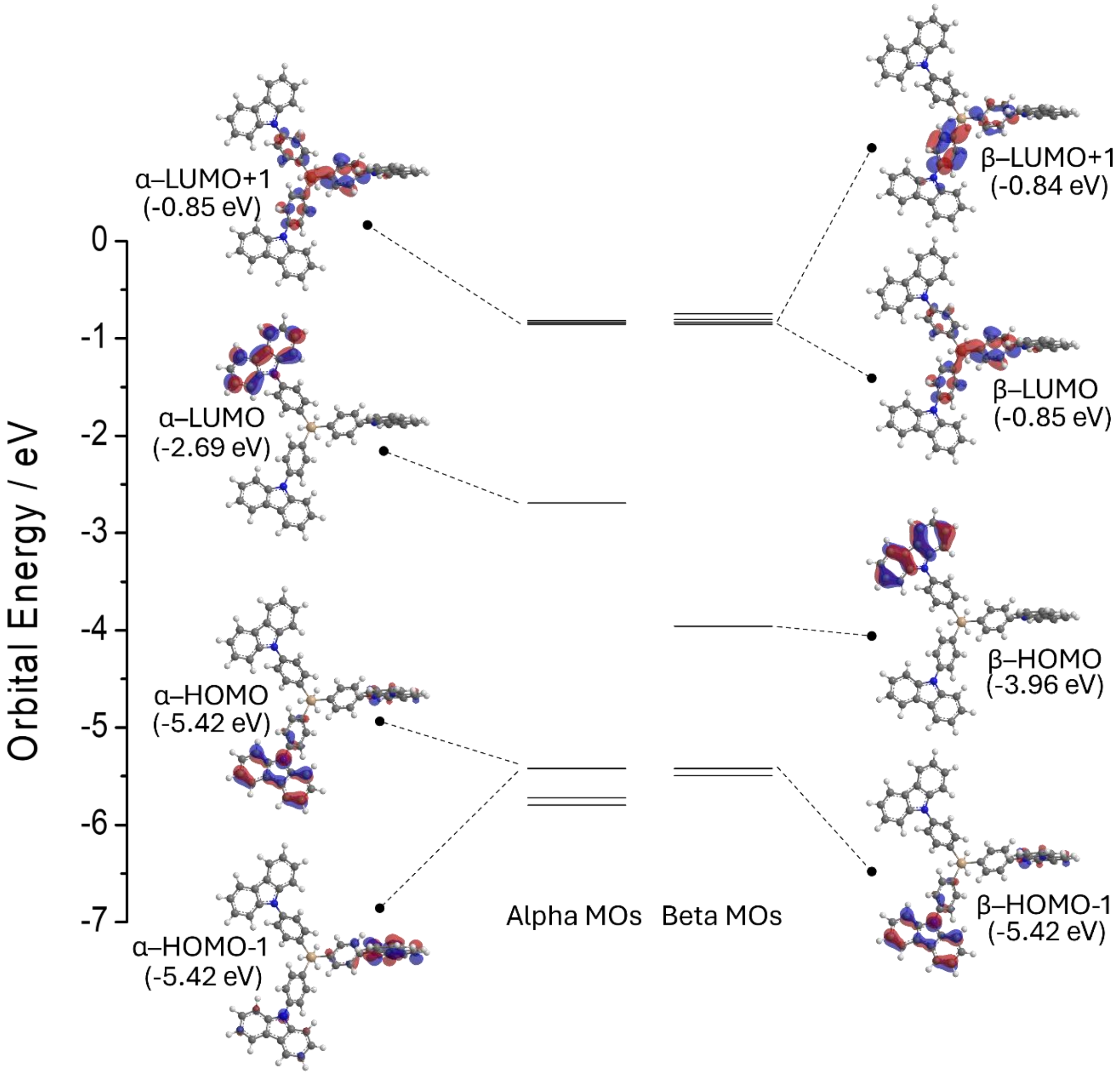

Figure S9. Frontier orbitals and orbital energy levels (isodensity contour = 0.04 a.u.) for selected occupied and unoccupied molecular orbitals of **MCBP-T$_0$**.

Table S10. Cartesian coordinates for optimized structure for **SiCBP4-S$_0$**.

Symbolic Z-matrix:
Charge = 0 Multiplicity = 1

Atom	X	Y	Z	Atom	X	Y	Z
Si	-5.9E-05	0.000119	0.000341	H	-5.22985	-5.30149	-4.44039
N	-4.98235	0.674806	3.532771	C	-3.18077	-4.97905	-3.90426
N	-0.67486	-4.97699	-3.53933	H	-3.38057	-4.07763	-3.33604
N	0.680218	4.981572	-3.53221	C	-1.88352	-5.48135	-4.03932
N	4.976573	-0.67987	3.539694	C	1.142721	2.542968	-0.76357
C	-1.51827	0.239818	1.104466	H	1.744399	2.455103	0.136625
C	0.241294	1.518149	-1.10367	C	1.284101	3.685503	-1.54869
C	1.516452	-0.24105	1.106452	H	1.967329	4.474918	-1.25348
C	-0.2397	-1.51651	-1.10596	C	0.532918	3.827453	-2.72209
C	-1.68467	-0.50673	2.287165	C	-0.36169	2.814181	-3.09061
H	-0.90743	-1.19648	2.605955	H	-0.92507	2.908478	-4.01305
C	-2.81392	-0.36495	3.091114	C	-0.50478	1.685143	-2.2866
H	-2.90776	-0.92869	4.013367	H	-1.19517	0.908535	-2.60555
C	-3.828	0.528795	2.722743	C	1.889531	5.486188	-4.03031
C	-3.68664	1.280467	1.549585	C	3.186345	4.98268	-3.89548
H	-4.47668	1.963034	1.254509	H	3.385135	4.079923	-3.32903
C	-2.5439	1.140407	0.76454	C	4.219623	5.676064	-4.522
H	-2.45652	1.742479	-0.13545	H	5.235989	5.304762	-4.42968
C	-5.81276	-0.36521	3.972651	C	3.97377	6.839187	-5.27084
C	-5.74024	-1.73512	3.705311	H	4.802042	7.356847	-5.7446
H	-4.95199	-2.14796	3.085961	C	2.678547	7.327034	-5.41459
C	-6.72173	-2.55643	4.255885	H	2.487614	8.219575	-6.00349
H	-6.68474	-3.62464	4.063624	C	1.621891	6.649072	-4.79601
C	-7.75634	-2.03213	5.048767	C	0.189685	6.858879	-4.75677
H	-8.50423	-2.70011	5.464591	C	-0.65689	7.832856	-5.29845
C	-7.83231	-0.66536	5.298815	H	-0.2499	8.639062	-5.902
H	-8.6389	-0.25925	5.902454	C	-2.02378	7.758163	-5.0487
C	-6.85915	0.182253	4.757292	H	-2.69097	8.5067	-5.46464
C	-6.65064	1.61464	4.796899	C	-2.54924	6.724012	-4.25599
C	-7.32945	2.670502	5.415914	H	-3.61752	6.688038	-4.06394
H	-8.22183	2.478613	6.004754	C	-1.72898	5.741719	-3.70529
C	-6.8427	3.966185	5.272595	H	-2.14271	4.953797	-3.08611
H	-7.361	4.793846	5.746715	C	-0.35894	5.812958	-3.97231
C	-5.67985	4.213289	4.523738	C	2.541695	-1.14256	0.767821
H	-5.3094	5.229998	4.431767	H	2.455019	-1.74432	-0.13244
C	-4.98564	3.180822	3.896805	C	3.683167	-1.2839	1.554489
H	-4.08314	3.380594	3.330284	H	4.47295	-1.9672	1.260418
C	-5.48803	1.883536	4.031228	C	3.823568	-0.53259	2.727991
C	0.507153	-1.68139	-2.28867	C	2.809842	0.362101	3.095055
H	1.19722	-0.90388	-2.60611	H	2.902922	0.925583	4.017547
C	0.365328	-2.80946	-3.09428	C	1.681886	0.505146	2.289515
H	0.929354	-2.90214	-4.01648	H	0.90491	1.195665	2.60729
C	-0.52879	-3.82384	-2.72762	C	5.480657	-1.88921	4.03827
C	-1.28071	-3.68401	-1.55444	C	4.977143	-3.18598	3.903039

H	-1.96349	-4.47435	-1.26066
C	-1.14061	-2.54242	-0.76772
H	-1.74285	-2.4562	0.132266
C	0.365159	-5.80658	-3.98073
C	1.734993	-5.73485	-3.71279
H	2.147773	-4.94788	-3.09178
C	2.556303	-6.7154	-4.26504
H	3.624456	-6.679	-4.07234
C	2.03206	-7.74834	-5.06013
H	2.700033	-8.49555	-5.47718
C	0.665362	-7.82357	-5.31077
H	0.259306	-8.62887	-5.91617
C	-0.18224	-6.8513	-4.76763
C	-1.61459	-6.64249	-4.80721
C	-2.67038	-7.31995	-5.42781
H	-2.47845	-8.21118	-6.01838
C	-3.96604	-6.83331	-5.28388
H	-4.79365	-7.35059	-5.75921
C	-4.21317	-5.67189	-4.53283
H	4.074979	-3.38473	3.335628
C	5.66978	-4.21928	4.530346
H	5.298453	-5.23561	4.437748
C	6.832159	-3.97349	5.280362
H	7.349231	-4.80177	5.754744
C	7.320002	-2.67831	5.424494
H	8.211981	-2.48742	6.01427
C	6.642795	-1.62163	4.80512
C	6.852701	-0.18944	4.766105
C	7.826183	0.657085	5.308761
H	8.631769	0.250039	5.913109
C	7.751815	2.023981	5.058946
H	8.499973	2.691123	5.475633
C	6.718496	2.54949	4.265187
H	6.682765	3.617779	4.073112
C	5.736716	1.729281	3.713488
H	4.949467	2.143056	3.093491
C	5.807608	0.359243	3.980587

Table S11. Cartesian coordinates for optimized structure for **SiCBP4-D_0**.

Symbolic Z-matrix:
Charge = 1 Multiplicity = 2

Atom	X	Y	Z	Atom	X	Y	Z
Si	0.000009	-6E-06	0.000014	H	-3.42451	-6.75411	4.194917
N	2.275289	-4.49373	-3.51204	C	-3.73718	-4.67946	3.755279
N	-4.4936	-2.27522	3.512264	H	-2.8232	-4.57265	3.18273
N	4.493682	2.275412	3.512047	C	-4.60678	-3.6021	3.95163
N	-2.27535	4.493526	-3.51224	C	2.06009	1.885964	0.732821
C	0.722652	-1.35916	-1.1016	H	1.800897	2.404336	-0.18591
C	1.359139	0.722685	1.101619	C	3.093813	2.397394	1.513134
C	-0.72266	1.359076	-1.10166	H	3.638623	3.279894	1.195469
C	-1.3591	-0.72263	1.101691	C	3.450348	1.756428	2.707398
C	0.097283	-1.73724	-2.30615	C	2.762273	0.600933	3.103375
H	-0.7886	-1.20783	-2.64684	H	3.015456	0.121599	4.042919
C	0.600859	-2.76228	-3.10336	C	1.737249	0.097324	2.306173
H	0.121523	-3.01543	-4.04291	H	1.207878	-0.78858	2.646856
C	1.75633	-3.45039	-2.70739	C	4.606823	3.602315	3.951353
C	2.397313	-3.09388	-1.51312	C	3.737206	4.679645	3.754918
H	3.279804	-3.63871	-1.19547	H	2.823248	4.572787	3.182339
C	1.885914	-2.06014	-0.73281	C	4.080238	5.900654	4.33312
H	2.404304	-1.80096	0.185917	H	3.424468	6.754307	4.194459
C	1.548125	-5.58663	-4.00473	C	5.250632	6.044346	5.0939
C	0.208404	-5.92825	-3.79564	H	5.490389	7.008304	5.530616
H	-0.44478	-5.31223	-3.18862	C	6.10364	4.959333	5.30298
C	-0.26005	-7.09967	-4.38824	H	6.999743	5.072807	5.905242
H	-1.29686	-7.38702	-4.24518	C	5.782798	3.726505	4.733417
C	0.582978	-7.91458	-5.15924	C	6.409068	2.417298	4.765196
H	0.188264	-8.82012	-5.60824	C	7.577981	1.924836	5.346859
C	1.92466	-7.57801	-5.34688	H	8.220074	2.575806	5.93213
H	2.575619	-8.22011	-5.93215	C	7.914572	0.58316	5.159211
C	2.417143	-6.40911	-4.7652	H	8.820125	0.188461	5.60821
C	3.726362	-5.78287	-4.73341	C	7.099673	-0.25989	4.388222
C	4.959186	-6.10373	-5.30297	H	7.387044	-1.29669	4.245159
H	5.072643	-6.99983	-5.90525	C	5.92824	0.208552	3.795633
C	6.044215	-5.25074	-5.09388	H	5.312229	-0.44464	3.188612
H	7.008171	-5.49051	-5.5306	C	5.586595	1.548266	4.004727
C	5.900544	-4.08036	-4.33309	C	-1.88594	2.060048	-0.73291
H	6.754209	-3.42461	-4.19442	H	-2.40433	1.800896	0.185824
C	4.679539	-3.73731	-3.75489	C	-2.39736	3.093741	-1.51327
H	4.572695	-2.82335	-3.1823	H	-3.27986	3.63857	-1.19564
C	3.602193	-4.6069	-3.95134	C	-1.75638	3.450221	-2.70754
C	-1.73715	-0.09724	2.306246	C	-0.60089	2.762118	-3.10348
H	-1.20776	0.788664	2.646889	H	-0.12154	3.015257	-4.04303
C	-2.76216	-0.6008	3.103491	C	-0.09729	1.737126	-2.30623
H	-3.01531	-0.12144	4.043032	H	0.788615	1.20774	-2.64688
C	-3.45028	-1.75629	2.707566	C	-3.60225	4.606655	-3.95155
C	-3.0938	-2.39729	1.513298	C	-4.67959	3.737061	-3.75506

H	-3.63865	-3.27978	1.195666	H	-4.57274	2.823138	-3.18243
C	-2.0601	-1.8859	0.732939	C	-5.90059	4.080072	-4.33329
H	-1.80095	-2.4043	-0.18578	H	-6.75425	3.424319	-4.19459
C	-5.5865	-1.54802	4.004912	C	-6.04427	5.250422	-5.09414
C	-5.92808	-0.2083	3.79578	H	-7.00823	5.490162	-5.53087
H	-5.31203	0.44486	3.188757	C	-4.95925	6.103408	-5.30327
C	-7.09952	0.260198	4.388325	H	-5.07271	6.999477	-5.90558
H	-7.38685	1.297006	4.245227	C	-3.72642	5.782587	-4.73368
C	-7.91447	-0.5828	5.159313	C	-2.41721	6.408845	-4.76549
H	-8.82002	-0.18806	5.608278	C	-1.92474	7.577721	-5.34722
C	-7.57793	-1.92448	5.347002	H	-2.5757	8.219786	-5.93253
H	-8.22006	-2.57542	5.932275	C	-0.58306	7.914315	-5.15957
C	-6.40902	-2.417	4.765381	H	-0.18835	8.81984	-5.60862
C	-5.78279	-3.72623	4.733661	C	0.259972	7.099457	-4.38853
C	-6.10368	-4.95903	5.303264	H	1.296774	7.386832	-4.24547
H	-6.9998	-5.07245	5.905503	C	-0.20848	5.928061	-3.79588
C	-5.25069	-6.04407	5.09426	H	0.444707	5.31208	-3.18882
H	-5.49048	-7.008	5.531009	C	-1.54819	5.586411	-4.00497
C	-4.08026	-5.90043	4.333519				

Table S12. Cartesian coordinates for optimized structure for **SiCBP4-T$_0$**.

Symbolic Z-matrix:
Charge = 0 Multiplicity = 3

Atom	X	Y	Z	Atom	X	Y	Z
Si	0.002869	0.0004	-0.001	H	-6.58291	-3.89897	4.224741
N	-0.305	-5.04491	-3.49528	C	-5.72316	-1.96448	3.708064
N	-5.10107	0.45334	3.387534	H	-4.88076	-2.38797	3.174786
N	4.907806	-0.38128	3.681094	C	-5.89213	-0.61497	3.839192
N	0.526442	4.966076	-3.58296	C	2.715003	0.561597	0.82994
C	-0.05712	-1.54492	-1.09229	H	2.789766	1.150812	-0.07966
C	1.504992	-0.08125	1.14792	C	3.83723	0.459064	1.6496
C	0.130474	1.521069	-1.12092	H	4.767753	0.942935	1.371837
C	-1.5604	0.105125	1.058017	C	3.773525	-0.28222	2.836076
C	-0.78159	-1.55683	-2.30001	C	2.575607	-0.92045	3.182768
H	-1.27449	-0.65138	-2.64449	H	2.514969	-1.47289	4.114614
C	-0.86194	-2.69793	-3.09571	C	1.466643	-0.82297	2.344592
H	-1.40113	-2.67637	-4.03699	H	0.546074	-1.3157	2.646528
C	-0.22345	-3.87828	-2.69353	C	5.654928	0.693812	4.181928
C	0.500513	-3.89226	-1.49482	C	5.457269	2.067601	4.017299
H	0.984138	-4.80896	-1.174	H	4.638027	2.452233	3.420403
C	0.586131	-2.73852	-0.71803	C	6.345551	2.931475	4.654173
H	1.161793	-2.77497	0.202513	H	6.212993	4.00321	4.539257
C	-1.4855	-5.63452	-3.96769	C	7.402853	2.446111	5.441984
C	-2.81493	-5.26416	-3.74672	H	8.0785	3.146584	5.92292
H	-3.06653	-4.39888	-3.14392	C	7.585426	1.077528	5.614914
C	-3.8114	-6.0499	-4.32171	H	8.395993	0.703145	6.233563
H	-4.85179	-5.78046	-4.16552	C	6.706832	0.187509	4.986599
C	-3.49872	-7.18148	-5.0936	C	6.593209	-1.25593	4.968993
H	-4.29929	-7.77105	-5.52944	C	7.338346	-2.28859	5.549636
C	-2.17365	-7.55419	-5.2976	H	8.197728	-2.06089	6.173751
H	-1.93295	-8.43554	-5.88514	C	6.967872	-3.60867	5.311959
C	-1.15298	-6.78239	-4.73074	H	7.536894	-4.41846	5.757942
C	0.291052	-6.88939	-4.72286	C	5.865198	-3.90437	4.492987
C	1.194161	-7.78687	-5.30405	H	5.597369	-4.94102	4.311179
H	0.832578	-8.62221	-5.89682	C	5.106314	-2.89571	3.903376
C	2.559201	-7.59111	-5.11805	H	4.263579	-3.13417	3.264375
H	3.269721	-8.28108	-5.56252	C	5.473812	-1.57151	4.157963
C	3.02814	-6.50224	-4.36401	C	-0.54399	2.718301	-0.82101
H	4.097452	-6.36013	-4.23844	H	-1.18277	2.771878	0.056011
C	2.151003	-5.59463	-3.77425	C	-0.41052	3.854274	-1.61708
H	2.523136	-4.75143	-3.20337	H	-0.92037	4.774689	-1.35241
C	0.780703	-5.80516	-3.95149	C	0.395438	3.817701	-2.76169
C	-1.59867	0.893499	2.224911	C	1.066519	2.632681	-3.09065
H	-0.70277	1.411288	2.557974	H	1.669805	2.592947	-3.99157
C	-2.75343	1.0086	2.995404	C	0.937019	1.509905	-2.27562
H	-2.75197	1.600577	3.904387	H	1.457044	0.599975	-2.56389
C	-3.9226	0.337853	2.610803	C	-0.5289	5.708551	-4.13055
C	-3.90948	-0.45179	1.453606	C	-1.90708	5.492654	-4.04413

H	-4.81547	-0.96277	1.145824	H	-2.31357	4.659874	-3.48144
C	-2.74302	-0.56766	0.700695	C	-2.74587	6.380817	-4.71385
H	-2.76041	-1.1915	-0.18849	H	-3.82056	6.234223	-4.6596
C	-5.67782	1.662161	3.823373	C	-2.23138	7.455772	-5.45809
C	-5.30392	2.954056	3.597618	H	-2.91289	8.130803	-5.96636
H	-4.43007	3.205898	3.009273	C	-0.85776	7.656654	-5.55332
C	-6.12618	4.001287	4.160862	H	-0.46025	8.480992	-6.13844
H	-5.84209	5.034529	3.992809	C	0.007482	6.778591	-4.8906
C	-7.27701	3.706087	4.919425	C	1.449112	6.681251	-4.7959
H	-7.8599	4.525456	5.330092	C	2.502641	7.447093	-5.30808
C	-7.67422	2.404277	5.149083	H	2.298758	8.313402	-5.93085
H	-8.55823	2.175189	5.733602	C	3.812266	7.0881	-5.0042
C	-6.87733	1.324205	4.585298	H	4.63808	7.673221	-5.39678
C	-7.01232	-0.04245	4.589497	C	4.076478	5.976325	-4.18677
C	-7.9891	-0.94447	5.178842	H	5.104894	5.717629	-3.95257
H	-8.83561	-0.54228	5.724147	C	3.046594	5.196897	-3.66449
C	-7.80646	-2.30072	5.028022	H	3.260194	4.347209	-3.0259
H	-8.52341	-2.991	5.463184	C	1.733816	5.553061	-3.98563
C	-6.7011	-2.82469	4.316644				

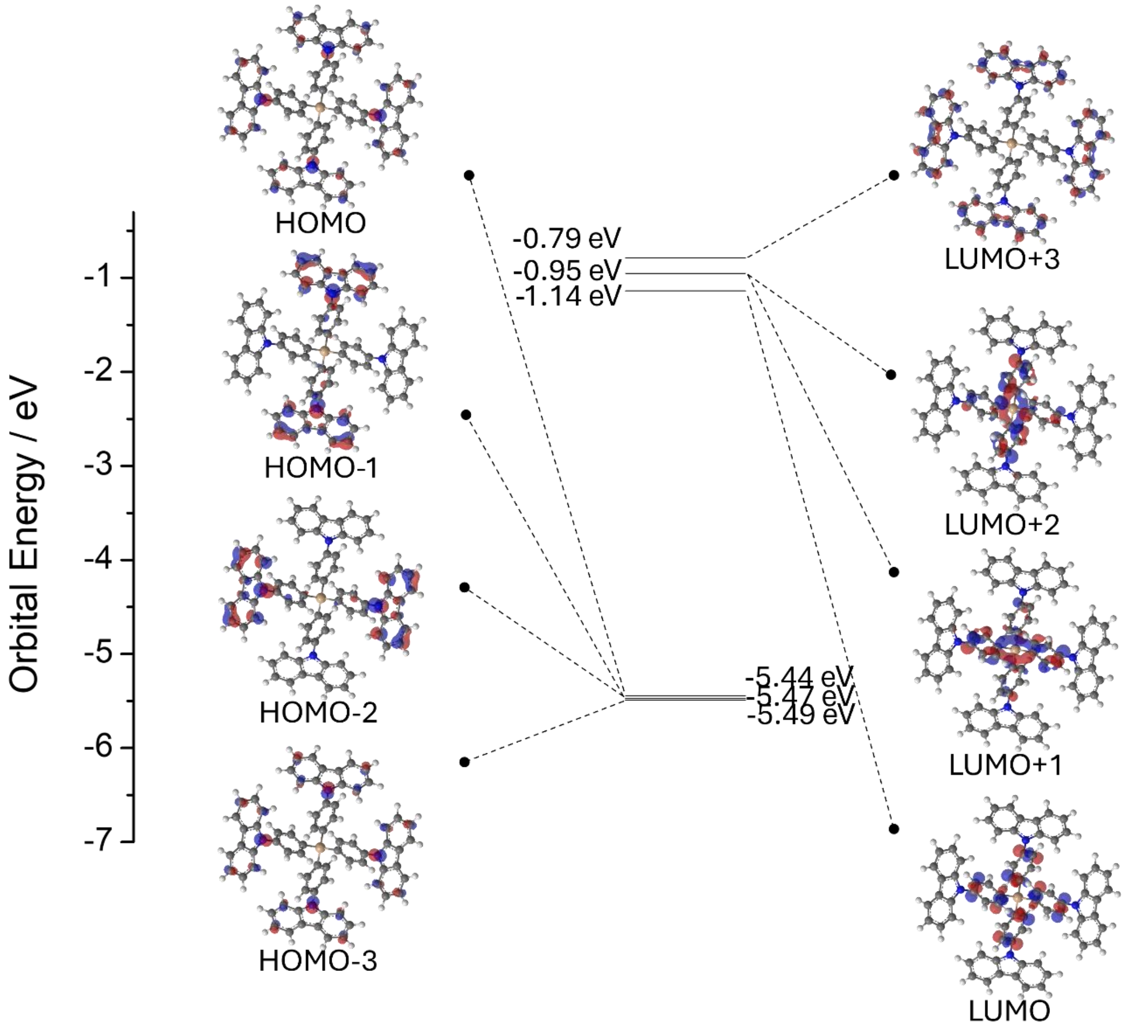

Figure S10. Frontier orbitals and orbital energy levels (isodensity contour = 0.04 a.u.) for selected occupied and unoccupied molecular orbitals of **SiCBP4-S$_0$**.

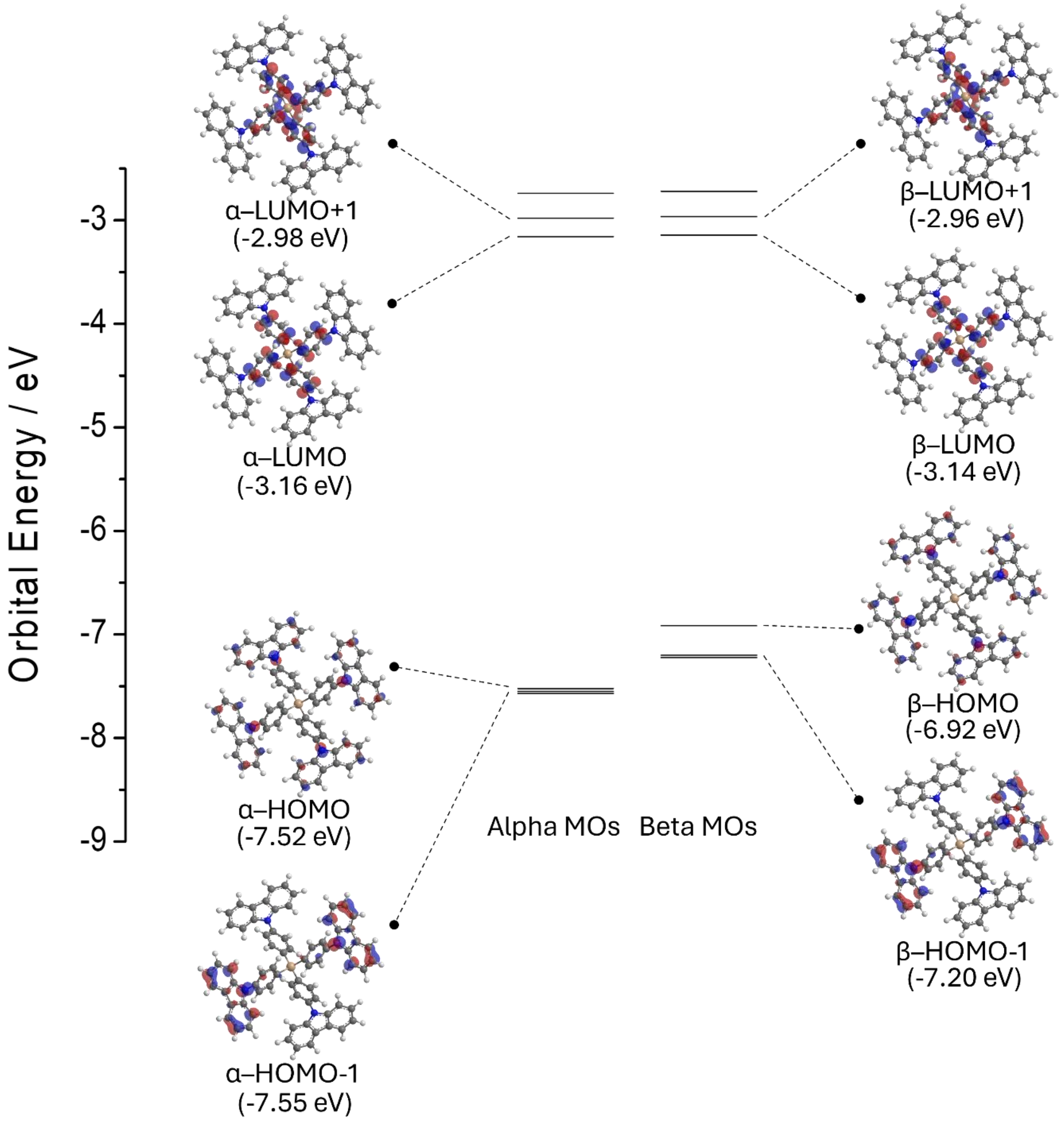

Figure S11. Frontier orbitals and orbital energy levels (isodensity contour = 0.04 a.u.) for selected occupied and unoccupied molecular orbitals of **SiCBP4-D$_0$**.

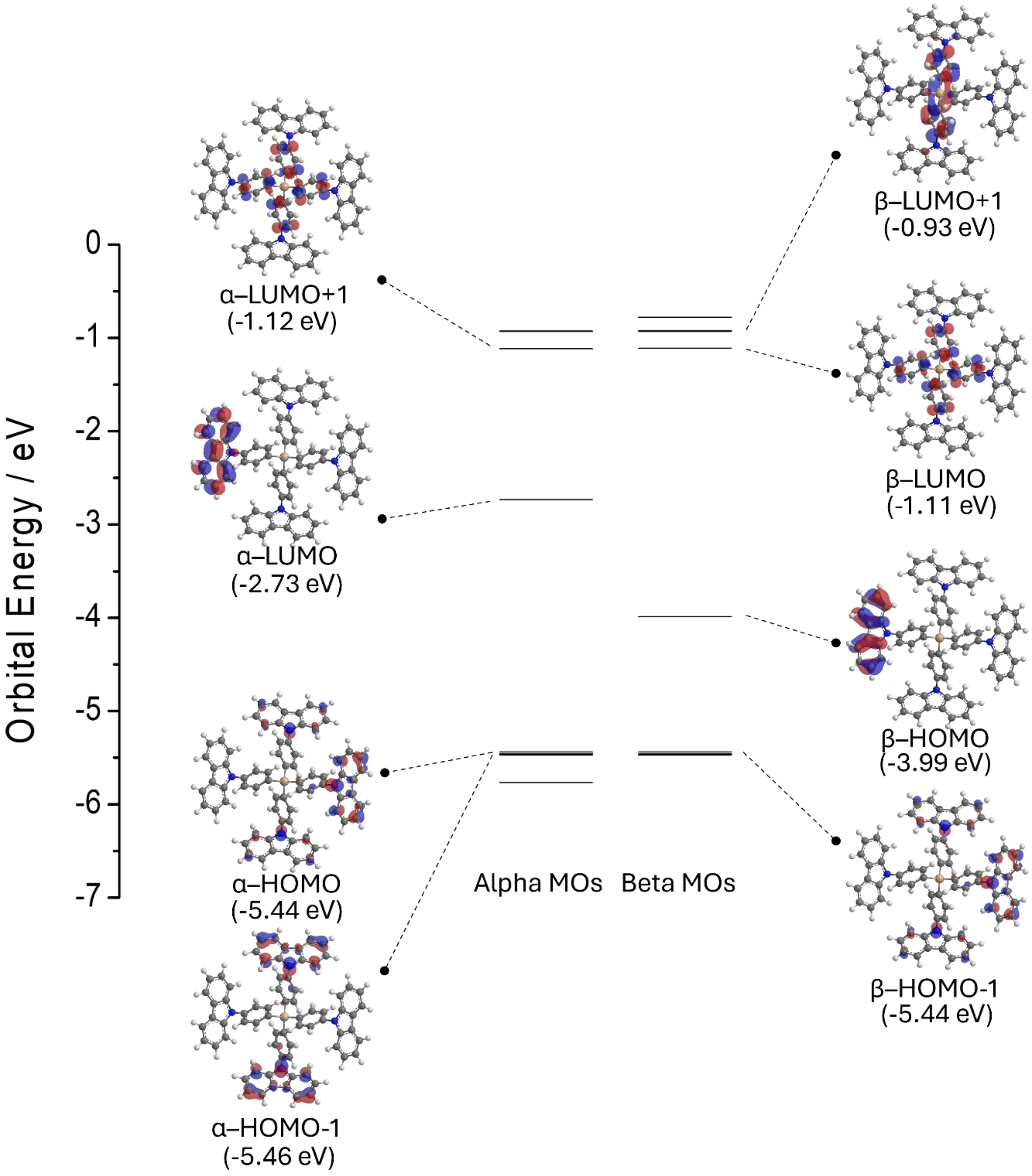

Figure S12. Frontier orbitals and orbital energy levels (isodensity contour = 0.04 a.u.) for selected occupied and unoccupied molecular orbitals of **SiCBP4-T$_0$**.

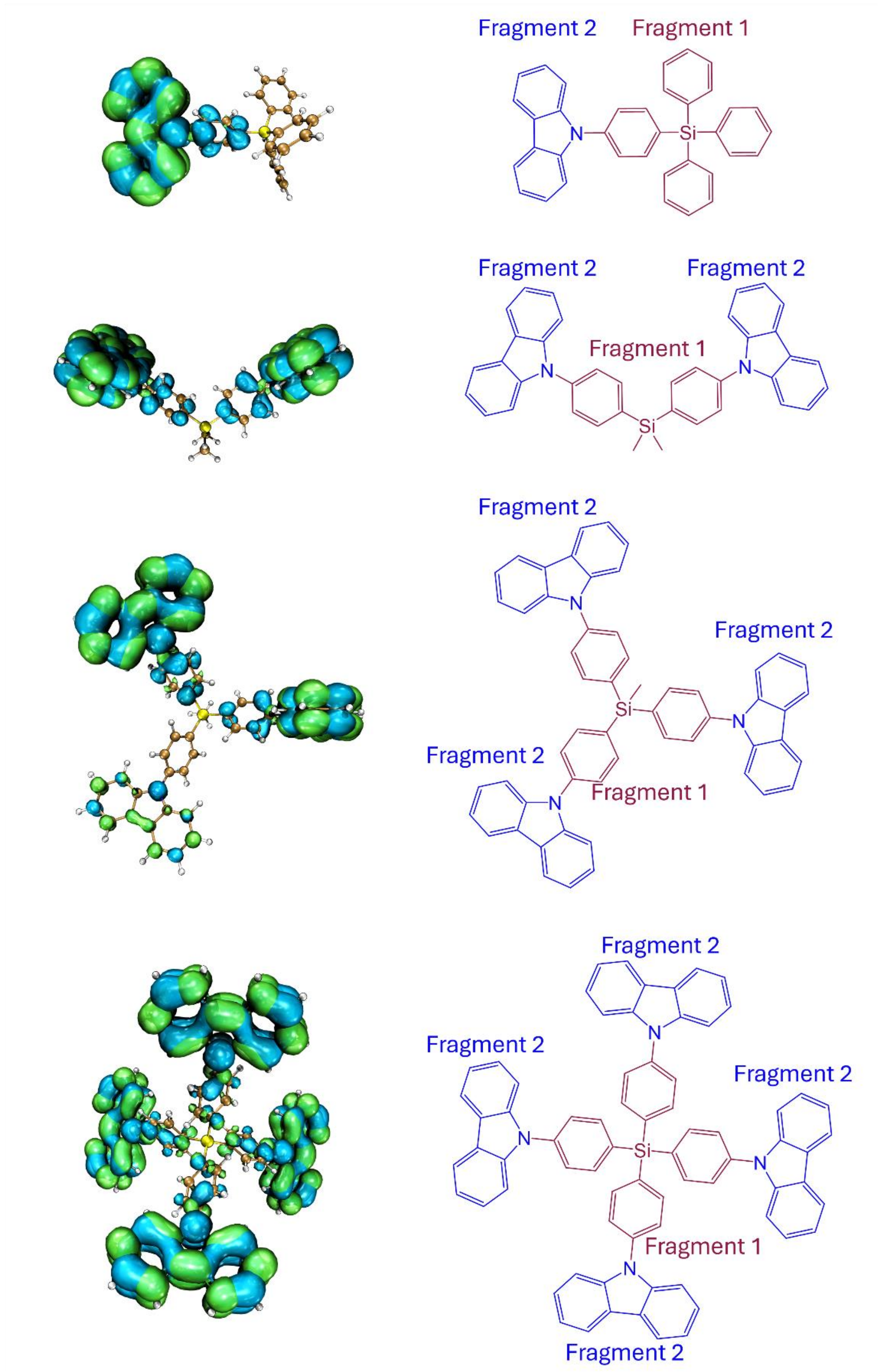

Figure S13. Hole and electron distribution for the $S_0 \rightarrow S_1$ transitions and two fragments in **3PCBP**, **2MCBP**, **MCBP**, and **SiCBP4**.

Table S13. Contributions of two fragments to hole, electron, and overlap of **3PCBP**, **2MCBP**, **MCBP**, and **SiCBP4**.

		Hole (%)	Electron (%)	Overlap (%)
**3PCBP**	1	6.15	1.42	2.96
	2	72.37	97.49	83.99
**2MCBP**	1	6.59	2.29	3.88
	2	93.41	97.71	95.54
**MCBP**	1	7.09	3.90	5.26
	2	92.91	96.10	94.49
**SiCBP4**	1	7.25	7.88	7.56
	2	92.75	92.12	92.43

Table S14. Physical properties of **3PCBP**, **2MCBP**, and **MCBP**

	T_1 (eV) [a]	E_{HOMO} (eV) [b]	E_{LUMO} (eV) [c]	Charge mobility (cm^2/Vs) [d]
**3PCBP**	3.01	-5.62	-2.60	1.32×10^{-4}
**2MCBP**	3.01	-5.37	-2.65	4.39×10^{-4}
**MCBP**	3.01	-5.42	-2.69	1.16×10^{-3}

[a] In 2-Methyltetrahydrofuran at 77 K [1].[b,c] The HOMO and LUMO levels were determined by DFT calculations.

[d] Under an electric field of 5×10^5 V/cm.

Reference

[S1] Zhang, M.; Ma, X.; Zhang, H.; Zhu, L.; Xu, L.; Zhang, F.; Tsang, C.-S.; Lee, L. Y. S.; Woo, H. Y.; He, Z., Metallated terpolymer donors with strongly absorbing iridium complex enables polymer solar cells with 16.71% efficiency. *Chem. Eng. J.* **2022**, *430*, 132832.
